# A role for antibiotic biosynthesis monooxygenase domain proteins in fidelity control during aromatic polyketide biosynthesis

**DOI:** 10.1038/s41467-019-11538-6

**Published:** 2019-08-09

**Authors:** Zhiwei Qin, Rebecca Devine, Matthew I. Hutchings, Barrie Wilkinson

**Affiliations:** 1 Department of Molecular Microbiology, John Innes Centre, Norwich Research Park, Norwich, NR4 7UH UK; 2 School of Biological Sciences, University of East Anglia, Norwich Research Park, Norwich, NR4 7TJ UK

**Keywords:** Bacterial genetics, Metabolic engineering, Natural product synthesis

## Abstract

The formicamycin biosynthetic gene cluster encodes two groups of type 2 polyketide antibiotics: the formicamycins and their biosynthetic precursors the fasamycins, both of which have activity against methicillin-resistant *Staphylococcus aureus*. Here, we report the formicapyridines which are encoded by the same gene cluster and are structurally and biosynthetically related to the fasamycins and formicamycins but comprise a rare pyridine moiety. These compounds are trace-level metabolites formed by derailment of the major biosynthetic pathway. Inspired by evolutionary logic we show that rational mutation of a single gene in the biosynthetic gene cluster encoding an antibiotic biosynthesis monooxygenase (ABM) superfamily protein leads to a significant increase both in total formicapyridine production and their enrichment relative to the fasamycins/formicamycins. Our observations broaden the polyketide biosynthetic landscape and identify a non-catalytic role for ABM superfamily proteins in type II polyketide synthase assemblages for maintaining biosynthetic pathway fidelity.

## Abstract (web summary)

Formicapyridines are similar to pentacyclic fasamycin and formicamycin aromatic polyketides but with a pyridine moiety. Here the authors rationally mutate the biosynthetic gene cluster to increase production and identify a non-catalytic role for the ABM superfamily of proteins.

## Introduction

An idealised linear biosynthetic pathway to a complex natural product can be imagined proceeding through a series of intermediate structures which would exist for some finite time as the pathway product accumulates. These hypothetical intermediates would exist freely in solution or bound to enzymes in the case of assembly line processes, and eventually all flux through the pathway would end and only the final product would exist. In reality no pathways proceed in this manner as the situation is complicated by varying rates of reaction for the different steps, meaning that some intermediates accumulate, while the inherent reactivity of other intermediates, or their ability to act as substrates for housekeeping enzymes not dedicated to the pathway, means that shunt metabolites often arise. Matters are further complicated by the fact that pathways are often convergent, with multiple units made in parallel before assembly into the final product, for example in the biosynthetic pathways to macrolide or aminoglycoside^1^ antibiotics. In addition, some pathways are not linear, and the final product is accessed via several routes due to the inherent substrate plasticity of the biosynthetic enzymes; well-studied examples include the rapamycin and erythromycin pathways^2^. Thus, in practice, any biosynthetic pathway will lead to the accumulation of a mixture comprising the final product plus varying concentrations of pathway intermediates and shunt metabolites. The composition of such a mixture will vary further when alternate growth conditions are used^3^. Sometimes the final product of the pathway cannot be clearly discerned.

**Fig. 1:**
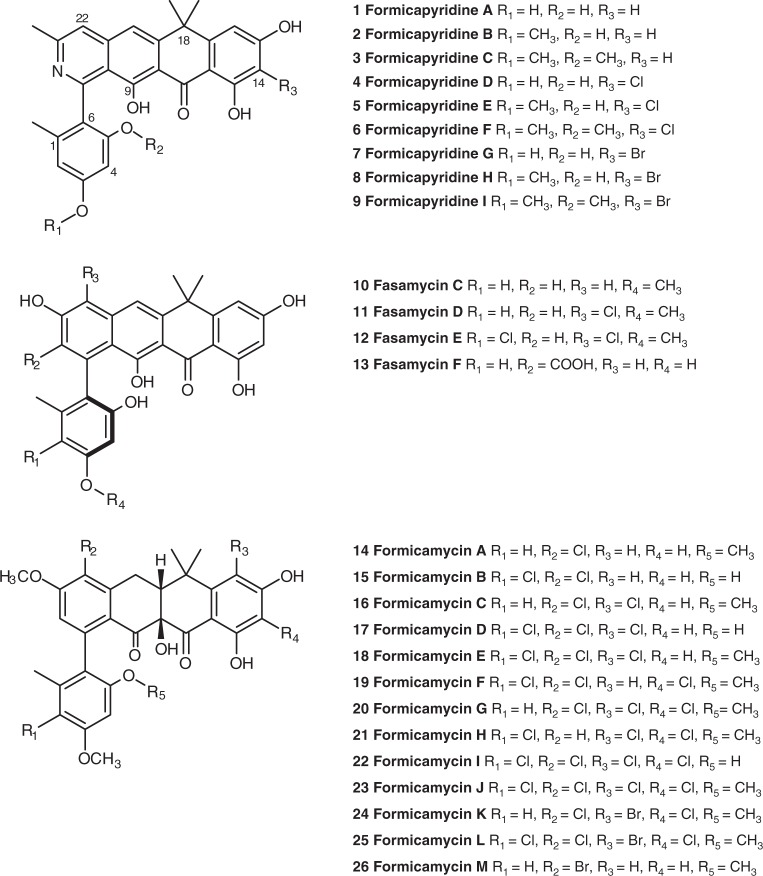
Chemical structures of metabolites isolated from *Streptomyces formicae*. Compounds **1**–**9** and **13** were discovered during this study whereas **10**–**12** and **14**–**26** were reported in an earlier study^5^

Such mixtures of compounds are said to comprise a series of biosynthetic congeners, and their identification can provide valuable information about a biosynthetic pathway and may sometimes lead to new biosynthetic understanding^4^. We recently reported the formicamycins, aromatic polyketides produced by *Streptomyces formicae* KY5 isolated from the fungus-growing plant-ant *Tetroponera penzigi* (Fig. 1)^5^. In total, sixteen congeners were isolated including three more fasamycins, a group of compounds previously reported from the heterologous expression of a clone derived from environmental DNA^6^. These congeners are the product of a type II polyketide synthase (PKS) operating in conjunction with post-PKS modifications that include *O*-methylation and halogenation, plus oxidative and reductive modifications. Intrigued by these compounds, which exhibit potent antibacterial activity, we employed targeted metabolomics to identify further congeners that may have been missed during manual analysis of culture extracts. This led us to identify the formicapyridines (**1**–**9**), pyridine containing polyketide alkaloids which represent additional products of the formicamycin (*for*) biosynthetic gene cluster (BGC). Products of type II PKS systems which contain a pyridine moiety are rare^7^.

Challis and co-workers have shown that the majority of actinomycete derived polyketide alkaloids, including those containing a pyridine moiety, arise from reactive intermediates formed after transamination of aldehydes generated from reductive off-loading of the thioester bound polyketide chain from a type I modular PK^8^. In contrast, the formicapyridines are minor shunt metabolites that arise due to derailment of the formicamycin biosynthetic pathway. We show here that reprogramming the *for* BGC, by deleting the *forS* gene encoding an ABM family protein, leads to significantly increased production of formicapyridines with concomitant reduction of the combined titre of fasamycins and formicamycins. These and other mutational data lead us to the hypothesis that ForS is not a cyclase but forms part of a multienzyme complex where it acts as a chaperone-like protein to aid in maintaining pathway fidelity and performance. The discovery and engineering of formicapyridine biosynthesis raises intriguing questions regarding the evolution of type II PKS biosynthetic pathways and the origins of natural product chemical diversity.

## Results

### Metabolomics led identification of the formicapyridines

**Fig. 2:**
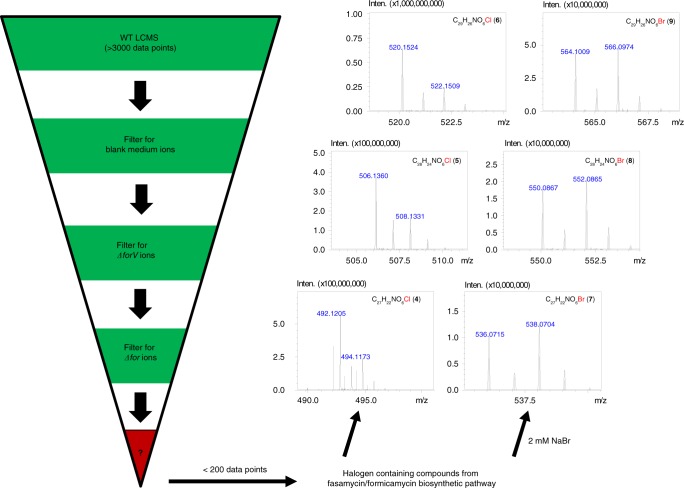
Metabolomics pipeline. Dereplication, based on *for* biosynthetic mutants and exogenous bromide addition, led to the identification of the new congeners **4–9** with characteristic halogen-containing patterns (strain *ΔforV* lacks a halogenase gene; *Δfor* lacks the entire biosynthetic gene cluster)

The aromatic, polycyclic nature of these molecules limited fragmentation and the effectiveness of molecular networking via the Global Natural Product Social Molecular Networking web platform^9^. Instead, as most fasamycin and formicamycin congeners are halogenated (Fig. 1), we established a bespoke dereplication method, making use of *S. formicae* KY5 mutants that we reported previously, including an entire BGC deletion strain (Δ*for*), and a strain in which the halogenase gene was deleted (Δ*forV*)^5^ (Fig. 2). Our earlier study^5^ showed that the Δ*for* mutant does not produce any fasamycins or formicamycins, and the Δ*forV* mutant produces only non-halogenated fasamycin congeners.

Replicate (*n* = 3) ethyl acetate extracts of the wild type (WT), Δ*for* and Δ*forV* strains, along with equivalent extracts from uninoculated mannitol soya flour (MS) agar plates, were analysed by liquid chromatography high-resolution mass spectroscopy (LC-HRMS). Using the Profiling Solutions software (Shimadzu Corporation), the WT dataset (Supplementary Data 1 and 2) was filtered to remove ions also present in the other samples. Only two other BGCs in the *S. formicae* KY5 genome^10^ contain putative halogenase encoding genes, so we hypothesised that any chlorine-containing ions present in the filtered dataset would likely derive from the formicamycin BGC. This process dramatically reduced the dataset complexity, leaving <200 unique ions from an original set of ∼3000. Manual curation showed that most of the remaining ions corresponded to halogenated molecules (based on isotope patterns), leaving twelve previously identified fasamycin and formicamycin congeners, two additional fasamycin/formicamycin congeners that remain uncharacterized due to trace levels, and the known isoflavone 6-chlorogenistein^11^ (Supplementary Note 1 and Supplementary Figs. 11–14) along with a regioisomer. Analysis of C/H ratios and *m/z* data allowed us to identify a group of three additional metabolites (**4**–**6**) with mass spectra suggesting a close structural relationship to the fasamycins/formicamycins; these varied only in the number of methyl groups present. By searching for equivalent ions lacking chlorine atoms we identified three additional congeners (**1**–**3**). Compounds **1**–**6** were not initially observed in the UV chromatograms of the WT, and qualitative examination of the LCMS data suggested titres at least 100-fold lower than for the formicamycins. Furthermore, the *m/z* data implied the presence of a single nitrogen atom.

We repeated the experiment but included a set of WT strain fermentations to which sodium bromide (2 mM) was added, as our previous study showed this leads to the biosynthesis of brominated formicamycin congeners^5^. This identified three conditional bromine-containing metabolites (**7**–**9**) with MS characteristics like those of **4**–**6** (Fig. 2 and Supplementary Fig. 1), supporting the hypothesis that these compounds represent biosynthetic congeners.

### Isolation and structure elucidation and biological activity

HRMS data allowed us to predict molecular formulae for **1**–**9** that were used to search the online chemical database REAXYS. This suggested that they represented new structures. To isolate sufficient material for structure determination and antibacterial assays, the growth of *S. formicae* KY5 was scaled up (14 L; ~450 MS agar plates). After 9 days incubation at 30 °C the combined agar was chopped up, extracted with ethyl acetate, and the solvent removed under reduced pressure. The resulting extract was subjected to repeated rounds of reversed-phase HPLC followed by Sephadex LH-20 size exclusion chromatography leading to small quantities of purified **1**–**6**.

**Fig. 3:**
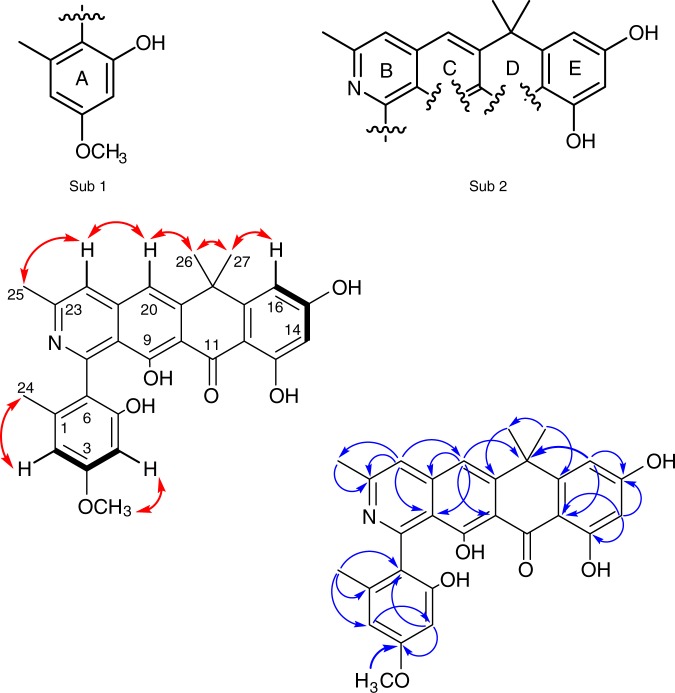
Structure determination. The COSY (black bold), NOESY (red double headed arrows) and HMBC (blue single headed arrows) correlations for formicapyridine B (**2**), along with the two resulting substructures are shown (Sub 1 and Sub 2)

We first determined the structure of **2** which was isolated in the largest amount (~2 mg). HRMS and ^13^C NMR data indicated a molecular formula of C_28_H_25_NO_6_ (calculated *m/z* 472.1755 ([M + H]^+^); observed *m/z* 472.1753 ([M + H]^+^); *Δ* = −0.4 ppm) indicating 17 degrees of unsaturation. The UV spectrum showed absorption maxima at 229, 249, 272 and 392 nm indicating a complex conjugated system different to that of the fasamycins/formicamycins^5^. Inspection of the ^13^C NMR spectrum showed 21 sp^2^ carbons (*δ*_C_ 99.90–167.54 ppm), one carbonyl carbon (*δ*_C_ 191.81 ppm), four methyl carbons (*δ*_C_ 20.41, 23.59, 34.56 and 37.75 ppm), one methoxy carbon (*δ*_C_ 55.80 ppm) and one sp^3^ quaternary carbon (*δ*_C_ 40.49 ppm). The ^1^H NMR spectrum revealed the presence of five methyl singlets (*δ*_H_ 1.75, 1.76, 1.93, 3.67 and 3.81 ppm), two aromatic proton singlets (*δ*_H_ 7.62 and 7.65 ppm), plus four aromatic proton doublets (*δ*_H_ 6.24 (d, 2.25 Hz), 6.70 (d, 2.25 Hz), 6.34 (d, 2.27 Hz) and 6.42 (d, 2.27 Hz)). Limited ^1^H–^1^H COSY correlations meant we were reliant upon HMBC-based atomic connections, and through-space NOESY correlations, which led to two potential substructures consisting of 26 carbon atoms, leaving one carbonyl, one phenol and one quaternary carbon unassigned (Fig. 3). This left some uncertainty, but given the relationship with the fasamycins, we predicted the structure to be that shown for **2**. We thus plotted the ^13^C chemical shifts for **2** against those for fasamycin C (**10**), the closest structural congener. The data were in excellent agreement with the main differences at C7, C22 and C23, consistent with the adjacent nitrogen atom (Supplementary Fig. 2). With the structure of **2** in hand we readily assigned the structures of **1** and **3**–**6**. NOESY correlations played a key role allowing us to link the methoxy group at C3 with H2 and H4 (e.g. **2**, **3**, **5** and **6**), and the methoxy at C5 with H4 (e.g. **3** and **6**). We also used NOESY and HSQC correlations to distinguish C14 and C16 once one was chlorinated: given the NOESY correlations to the *gem*-dimethyl groups of C26 and C27 with H16, and the disappearance of a HSQC linkage for C14, we concluded that C14 was chlorinated (e.g. **4**–**6**). Compounds **1**–**6** exhibit optical activity with small [*α*]^20^_D_ values between +8° and +13° indicating probable atropoisomerism about the axis of the C6–C7 bond. We attempted to assign the absolute configuration by combining computational approaches with electronic circular dichroism (ECD) spectra as done for the fasamycins^5^. However, despite scaling up production to isolate additional **2**, meaningful ECD spectra could not be obtained. Full characterization data for **1**–**6** are presented in Supplementary Note 1. Due to the very low levels of **7**–**9**, we have assigned preliminary structures based on **4**–**6**, with a bromine atom replacing chlorine at C14.

Compounds **1**–**6** displayed no antibacterial activity against *Bacillus subtilis* 168^12^ using overlay assays at 5 µg mL^−1^ (the average concentration for illustrating fasamycin and formicamycin bioactivity). Similarly, no inhibition was seen at tenfold this concentration (50 µg mL^−1^), with only small zones of inhibition at 100-fold (500 µg mL^−1^). To confirm this was not a result of reduced diffusion from the disc, assays were set up to test growth of *B. subtillis* 168 in liquid cultures containing compounds **1**–**6**, but again they showed no activity.

### Biosynthetic origins

Interrogation of LC-HRMS data from extracts of the Δ*for* and Δ*forV* mutants verified that the fasamycin/formicamycin BGC is required for formicapyridine production. The Δ*for* mutant does not produce **1**–**6** (Supplementary Fig. 3b vs. 3a), but production of all six compounds was restored upon ectopic expression of the P1-derived artificial chromosome (PAC) pESAC13–215-G containing the entire formicamycin BGC (Supplementary Fig. 3c). Similarly, the ∆*forV* mutant does not produce **4**–**6** (Supplementary Fig. 3d), but their production is re-established upon ectopic expression of *forV* under the control of the native promoter using an integrative plasmid (Supplementary Fig. 3e) (the construction of all strains, the requirement of the *for* BGC for production of the fasamycins and formicamycins, and the requirement of ForV for their halogenation were described previously^5^).

**Fig. 4:**
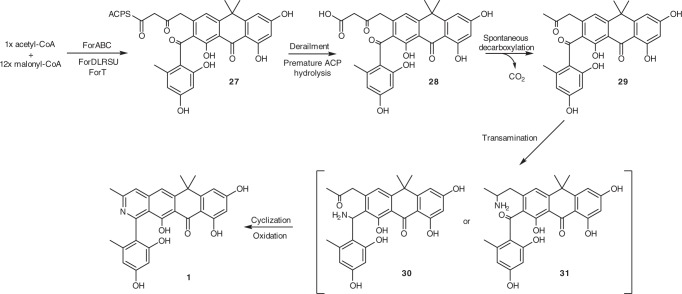
Proposed biosynthesis of the formicapyridine backbone. Derailment of fasamycin biosynthesis due to premature hydrolysis of the PKS acylcarrier protein leads to the β-ketoacid intermediate **28** which is facile to decarboxylation yielding methylketone **29**. Transamination of **29** leads of amine intermediates which can undergo spontaneous cyclization and aromatization to yield formicapyridines

The very low level of formicapyridines made by the WT strain suggests they are shunt metabolites arising from aberrant derailment of fasamycin/formicamycin biosynthesis. On this basis, we suggest a biosynthetic pathway as described in Fig. 4. Assembly of the poly-β-ketone tridecaketide intermediate should proceed as previously proposed on route to the fasamycins^5^. This would be followed by a series of cyclization and aromatisation steps, presumably in a sequential manner, with the final cyclization event probably involving formation of the B-ring. However, premature hydrolysis of the acylcarrier protein from putative intermediate **27**, prior to the action of a final cyclase, would liberate the enzyme-free, β-ketoacid species **28** that would be highly facile to spontaneous decarboxylation yielding the methylketone **29**. The action of an endogenous aminotransferase could then generate either of the species **30** or **31**, which could undergo cyclization, dehydration and oxidation to yield **1**. Consistent with a shunt pathway, no aminotransferase encoding gene could be identified in the *for* BGC, and although numerous putative aminotransferase encoding genes were identified in the genome, none could be linked to a role in formicapyridine biosynthesis. Alternatively, direct addition of ammonia to the C23 ketone would generate an imine, tautomerize to the enamine, and the resulting amino group could then attack the C7 ketone. Subsequent dehydration would lead directly to the formicapyridine system with no requirement of an oxidation reaction.

**Fig. 5:**
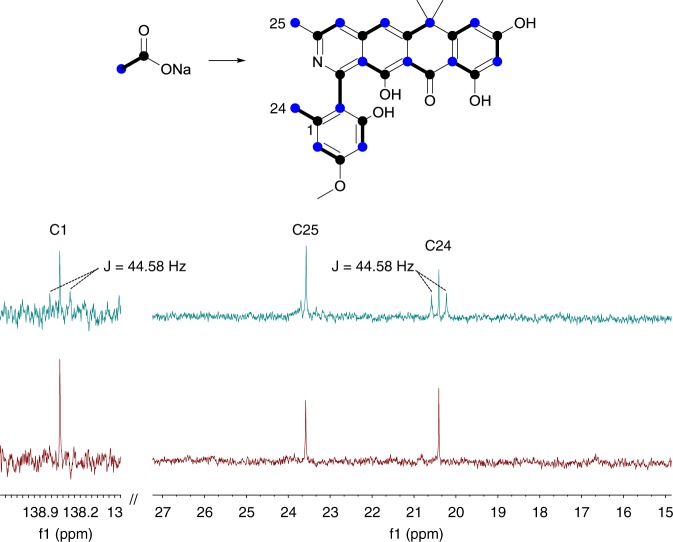
The methyl group carbon C25 arises from C2 of acetate. Comparison of ^13^C NMR spectra for **2** isolated after growth in the presence of [1,2-^13^C_2_] sodium acetate. Integration of the ^13^C NMR spectra confirmed weak enrichment of the ^13^C isotope and the absence of coupling to any adjacent carbon atom. In contrast, the methyl group atom C24 shows enrichment and coupling to C1. The C2 atom of [1,2-^13^C_2_] sodium acetate is highlighted as a blue circle, the C1 atom as a black circle, and the coupled unit by a bold line

This pathway requires that C25 of the formicapyridines originates from C2 of an acetate unit, with C1 lost via decarboxylation. To support our hypothesis, and the backbone structural assignments, *S. formicae* KY5 was cultivated on MS agar plates for two days and then overlaid with a solution of [1,2-^13^C_2_] sodium acetate (1 mL of 60 mM solution; final concentration 2 mM). This was repeated on the four following days, and after a total of 9 days incubation the agar was extracted with ethyl acetate and the most abundant congener was isolated (**2**, ~1 mg), and the ^13^C NMR spectrum was acquired. Due to the small amount of material, and overlapping signals, only eight of the intact acetate units could be unambiguously identified based on their coupling contests. In addition, a singlet was observed for C25 which showed weak enrichment (Fig. 5; Supplementary Fig. 4). This is consistent with our biosynthetic hypothesis, which requires that the carbon atom at C25 derives from C2 of a fragmented acetate unit. The data were in accordance with the proposed structure for **2**.

### Targeted evolution of the *for* BGC

Our biosynthetic proposal led to the following thought experiment. Suppose, in some environmental scenario, the presence of formicapyridines leads to a selection advantage. Is there then a single mutation in the BGC that could rapidly lead to enhanced levels of their production, and, in addition, could such changes lead to the reduction or even abolition of fasamycin/formicamycin biosynthesis?

The proposed biosynthetic pathway likely requires the action of multiple polyketide cyclase/aromatase enzymes and this suggests that one cyclase could be dedicated to formation of ring-B in the final step of backbone biosynthesis (Fig. 4). On this basis we hypothesised that mutation of a gene encoding a putative ring-B cyclase might lead to the phenotype imagined in our thought experiment by eliminating the biosynthesis of fasamycins/formicamycins and shunting carbon flux into the proposed formicapyridine pathway. BLAST and conserved domain analysis, in conjunction with structural modelling using the Phyre2 web portal for protein modelling and analysis^13^, allowed us to identify five genes in the *for* BGC, which might encode potential cyclase/aromatase enzymes (Table 1).

**Table 1:** Characteristics of the putative PKS cyclase genes in the *for* BGC

Name	Sequence identity	Top BLASTp hit	Annotation	% Coverage	% Identity
ForD	WP_098245758.1	WP_003962252.1	Polyketide cyclase (*TcmN*), *Streptomyces clavuligerus* ATCC 27064	95	73
ForL	WP_055544278.1	WP_076971949.1	*TcmI* family type II polyketide cyclase, *Streptomyces sparsogenes*	97	52
ForR	WP_098245762.1	AUI41024.1	Polyketide WhiE II *Streptomyces* sp., cupin domain	96	75
ForS	WP_098245763	SDF47214.1	Antibiotic biosynthesis monooxygenase, *Lechevalieria fradiae*	96	48
ForU	WP_098245765.1	PZS28546.1	Antibiotic biosynthesis monooxygenase, *Pseudonocardiales* bacteria	84	46

The gene product ForD shows significant sequence similarity to aromatase/cyclases such as the N-terminal domain (pfam 03364) of the archetypical tetracenomycin polyketide cyclase TcmN, which belongs to the Bet v1-like superfamily (cl10022)^14^. In vivo analysis^15–17^ and in vitro reconstruction^18^ showed that TcmN catalyses formation of the first two rings of tetracenomycin via sequential C9–C14 and C7–C16 cyclization/aromatization reactions. Thus, we predict ForD, which will play a key role in formation of the rings E and D during fasamycin biosynthesis. The ForL gene product belongs to the TcmI family of polyketide cyclases (cl24023; pfam 04673). The function of TcmI has been verified by in vivo mutational analysis^19^ and biochemical characterisation^20,21^. It catalyses cyclization of the final ring during formation of tetracenomycin F1 from tetracenomycin F2, and this reaction is remarkably like that proposed in our hypothesis for the formation of ring-B as the final step of fasamycin backbone assembly (Fig. 4). ForR is a homologue of the zinc containing polyketide cyclase RemF from the resistomycin BGC^22,23^. RemF is a single domain protein (pfam 07883) comprising the conserved barrel domain of the cupin superfamily (cl21464)^24^. Finally, the gene products ForS and ForU are both single domain proteins (pfam 03992) belonging to the ABM superfamily (cl10022). A notable member of this family is ActVA-orf6, which functions as a monooxygenase during biosynthesis of the polyketide antibiotic actinorhodin; the structure of this enzyme has been solved and it is topologically related to the PKS cyclase TcmI and homologues^25^. Notably, ABM family domains are found in several PKS cyclases including the C-terminal domain of BenH from the benastatin BGC^26,27^, which also comprises an N-terminal TcmN like cyclase domain (pfam 03364); and WhiE Protein 1 and other members of the SchA/CurD-like family of PKS enzymes commonly associated with BGCs for the biosynthesis of spore pigments in *Streptomyces* spp^28–30^. SchA/CurD- and WhiE Protein 1-like enzymes are comprised of an N-terminal ABM domain and a C-terminal PKS cyclase domain (pfam 00486; superfamily cl24023).

To interrogate their roles, we used Cas9-mediated genome editing^31^ to make in-frame deletions in each of these five putative cyclase genes (*forD*, *forL*, *forR*, *forS* and *forU*). Three independent mutants generated from each gene deletion experiment, along with the WT strain, were grown on MS agar and cultured at 30 °C for 9 days. To assess differences in secondary metabolite production the ethyl acetate extracts from each culture were subjected to HPLC(UV) and LCMS analysis (Supplementary Data 3 and 4). The metabolic profiles showed that the *ΔforD*, *ΔforL*, *ΔforR* and *ΔforU* mutants lost the ability to produce formicapyridines, fasamycins and formicamycins (Supplementary Figs. 5–9), and no new shunt metabolites could be identified despite rigorous interrogation of the LCMS and LC(UV) data. These deletions could be rescued by complementation with the deleted gene under control of either the native promoter (Δ*forD/forD* and Δ*forL/forL*) or the constitutive ermE* promoter (Δ*forR/forR*), although the titres did not reach that of the WT strain in all cases. For the Δ*forU* mutant complementation with *forU* restored production of the non-halogenated congener **10** only, indicating a polar effect on the downstream halogenase *forV* gene. Subsequent complementation with a *forUV* cassette under the ermE* promoter in which the two genes were transcriptionally fused led to the production of halogenated fasamycin and formicamycin congeners.

**Fig. 6:**
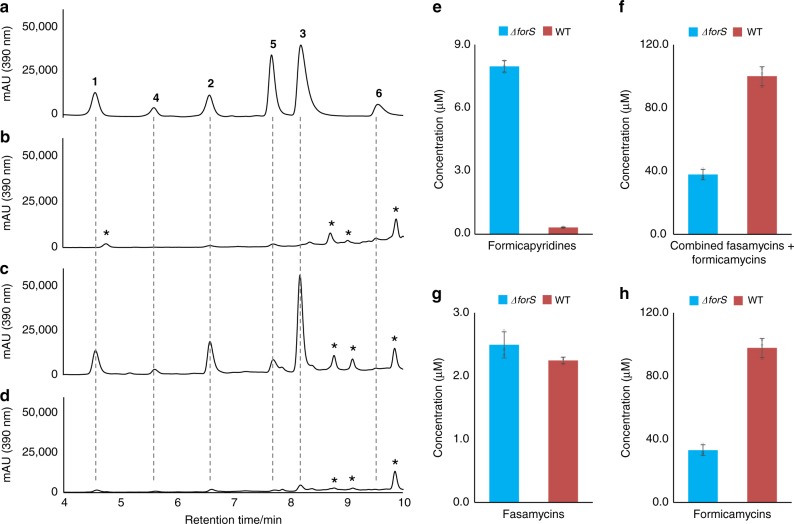
Mutational analysis of *forS*. Reconstituted HPLC chromatograms (UV; 390 nm) showing: **a** formicapyridine standards **1**–**6**; **b** *S. formicae* WT extract; **c** *S. formicae ∆forS* extract; **d** *S. formicae ∆forS/forS* extract. Quantitative data for the combined titre of each metabolite family produced by the *S. formicae* WT and *∆forS* mutants are shown for: **e** total formicapyridines; **f** combined total fasamycins and formicamycins; **g** total fasamycins; **h** total formicamycins; (mean ± standard deviation; *n* = 3 (biological replicates)). The molecular species giving rise to peaks labelled with an asterisk are unrelated to the *for* pathway based on UV and *m/z* analysis. The source data underlying Fig. 6e–h are provided as a Source Data file

In contrast, production of formicapyridines was increased ∼25-fold in the Δ*forS* mutant when compared with the WT (Fig. 6). These effects were complemented by ectopic expression of *forS* under the control of the ermE* promoter. Moreover, the Δ*forS* mutant was significantly compromised in its ability to produce formicamycins, with their titre being reduced to approximately one third that of the WT strain. This is consistent with our hypothesis for formicapyridine biosynthesis and suggests that ForS plays a role during B-ring closure, and that this constitutes the final step of fasamycin backbone biosynthesis. It also demonstrates that the final cyclization step can still occur without ForS. While carrying out analysis of the Δ*forS* mutant we identified a minor congener, which was not identified in any WT strain fermentations. Scale up growth (4 L) and solvent extraction, followed by isolation (3.4 mg) and structural elucidation identified this compound (**13**) as the C24-carboxyl analogue of fasamycin C (which we have named fasamycin F). This is the first congener identified with the C24-carboxyl group intact.

## Discussion

Using targeted metabolomics, we identified a family of pyridine containing polyketide natural products that we have named the formicapyridines. Remarkably, these compounds are derived from the fasamycin/formicamycin biosynthetic machinery meaning that the *for* BGC is responsible for the production of three structurally differentiated pentacyclic scaffolds. Inspired by evolutionary considerations we introduced a gene deletion into the BGC, which significantly altered the relative levels of these metabolites: the titre of formicapyridines was significantly increased (∼25-fold) in contrast to the fasamycin/formicamycins, which were decreased to approximately one-third of the WT titre. These results raise a series of questions about the cyclization events associated with the fasamycin/formicamycin biosynthetic pathway, and with the maintenance of pathway fidelity.

Our data suggest that formation of ring-B of the fasamycin scaffold is the final biosynthetic step, followed by thioester hydrolysis to liberate the ACP and subsequent decarboxylation to remove the carboxyl group attached to C24 (Fig. 5). Consistent with this, deletion of *forS* led to the isolation of shunt metabolite **13** with a C24-carboxyl group that was not observed from the WT. However, while ForS is implicated in ring-B cyclization it is not required for this role, nor, apparently, for the biosynthesis of any congeners. Rather, ForS seems to decrease the production of aberrant congeners from the pathway, e.g. formicapyridines, while increasing overall productivity. The most parsimonious interpretation of these data is that another of the *for* BGC gene products is the actual catalyst for ring-B formation, and that ForS acts as a chaperone, which modulates or stabilises the assembly, or arrangement, of a multienzyme complex to optimise production of the fasamycins, and therefore ultimately the formicamycins. This has the consequence of minimising the production of shunt metabolites (formicapyridines). Thus, while deletion of *forS* leads to the phenotype desired from our thought experiment, the mechanism by which this occurs is not what was anticipated. Interpretation of the *for* BGC bioinformatic analysis suggests the most likely candidate for a ring-B (final) cyclase is ForL due to its close relationship with TcmI, which catalyses a similar final cyclization step during tetracenomycin biosynthesis^19–21^. The apparent lack of any intermediates or shunt metabolites being accumulated by the remaining mutants is somewhat surprising and suggests an absolute requirement for the formation of a PKS-cyclase complex before biosynthesis can be initiated.

These observations are reminiscent of studies regarding the biosynthesis of pradimicin^32^. Pradimicins are pentangular polyketides similar to the benastatins^26,27^, and the BGC for their production contains three PKS cyclases equivalent to ForD (PdmD), ForL (PdmK) and ForR (PdmL)^32,33^. It also contains two ABM domain proteins (PdmH and PdmI; c.f. ForS and ForU) which were assigned monooxygenase roles. Through heterologous expression of PKS gene cassettes it was deduced that for the biosynthetic pathway to function correctly, and yield a pentangular backbone, all three cyclase genes (PdmDKL) plus the ABM domain monooxygenase PdmH must be co-expressed. In this pathway one oxidation reaction to form the quinone structure is required. These results led the authors to propose a model in which the two cyclases PdmKL and the monooxygenase PdmH form a multienzyme complex that engulfs the entire polyketide molecule during its assembly and work synergistically to ensure the correct reaction pathway occurs thereby minimising the production of shunt metabolites^32^. Similarly, formation of the unusual discoid metabolite resistomycin involves an extremely rare S-shaped folding pattern and requires the coordinated function, likely as a multienzyme complex, of core PKS proteins in addition to three distinct cyclase enzymes^23^. Moreover, heterologous reconstruction of the resistomycin pathway gave no products when the minimal PKS plus first cyclase were assembled^23^, a rare observation that is in keeping with our data showing the requirement of all the putative cyclases ForDLRU to produce any pathway derived metabolites, including shunts.

Biosynthesis of the *for* BGC polyketide backbones does not require a monooxygenase. Consistent with this, deletion of *forS* does not abolish polyketide production, but instead affects pathway productivity and fidelity. Thus, based on our mutational data and the observations discussed above, we hypothesise that ABM family enzymes can act as monooxygenases and/or as ancillary proteins to tune, in some way, the PKS enzyme complex function, and therefore the biosynthetic pathway, during aromatic polyketide biosynthesis. Genes encoding these proteins occur commonly in PKS BGCs, and multiple paralogues are often present even when monooxygenase reactions are not required. It is noteworthy that several PKS cyclases occur as fusion proteins with an ABM domain, which can be located at either terminus. We speculate that these may represent examples of mature pathways where the chaperone-like function of the ABM family protein has become essential, leading to selective pressure for the encoding gene to become transcriptionally fused with other cyclase encoding genes.

## Methods

### Chemistry methods and materials

Unless stated otherwise all chemicals were supplied by Sigma-Aldrich or Fisher Scientific. [1,2-^13^C_2_] sodium acetate was purchased from CORTECNET. All solvents were of HPLC grade or equivalent. NMR spectra were recorded on a Bruker Avance III 400 MHz NMR spectrometer equipped with 5 mm BBFO Plus probe. ^13^C NMR spectra for **2** and isotopically enriched **2** after feeding [1,2-^13^C_2_] sodium acetate were recorded on a Bruker Avance III 500 MHz NMR spectrometer equipped with a DUL cryoprobe at 30 °C.

Unless otherwise stated samples were analysed by LCMS/MS on a Nexera/Prominence UHPLC system attached to a Shimadzu ion-trap time-of-flight mass spectrometer. The spray chamber conditions were: heat block, 300 °C; 250° curved desorbtion line; interface (probe) voltage: 4.5 KV nebulizer gas flow rate 1.5 L min^−1^; drying gas on. The instrument was calibrated using sodium trifluoroacetate cluster ions according to the manufacturer’s instructions and run with positive–negative mode switching. The following analytical LCMS method was used throughout this study unless otherwise stated: Phenomenex Kinetex C_18_ column (100 × 2.1 mm, 100 Å); mobile phase A: water +0.1% formic acid; mobile phase B: methanol. Elution gradient: 0–1 min, 20% B; 1–12 min, 20–100% B; 12–14 min, 100% B; 14–14.1 min, 100–20% B; 14.1–17 min, 20% B; flow rate 0.6 mL min^−1^; injection volume 10 µL. Samples were prepared for LCMS analysis by taking a rectangle of agar (2 cm^3^) from an agar plate culture and shaking with ethyl acetate (1 mL) for 20 min. The ethyl acetate was transferred to a clean tube and the solvent removed under reduced pressure. The resulting extract was dissolved in methanol (200 µL).

### Standard microbiology and molecular biology methods

All strains used or made in this study are described in Supplementary Table 1. All plasmids and ePACs used are described in Supplementary Table 2. All PCR primers used are described in Supplementary Data 5. The compositions of media used are described in Supplementary Table 3. The antibiotics and their concentrations used are described in Supplementary Table 4. Standard DNA sequencing was carried out by Eurofins Genomics using the Mi×2Seq kit (Ebersberg, Germany).

*E. coli* strains were cultivated at 37 °C in LB Lennox Broth (LB), shaking at 220 rpm, or LB agar supplemented with antibiotics as appropriate. *S. formicae* was cultivated at 30 °C on MS agar or MYM agar with appropriate antibiotic selection. To prepare *Streptomyces* spores, material from a single colony was plated out using a sterile cotton bud and incubated at 30 °C for 7–10 days until a confluent lawn had grown over the entire surface of the agar. Spores were harvested by applying 20% glycerol (2 mL) to the surface of the agar plate culture and gently removing spores with a sterile cotton bud before storing them at −80 °C. Glycerol stocks of *E. coli* were made by pelleting the cells from an overnight *E. coli* culture (3–5 mL) in a bench top centrifuge (2773 × g, 5 min) and resuspending in fresh, sterile 1:1 2YT/40% glycerol (1 mL). Glycerol stocks were stored at −80 °C.

*S. formicae* genomic DNA and cosmid ePAC DNA (from *E. coli* DH10B) was isolated using a phenol:chloroform extraction method. Briefly, cells from an overnight culture (1 mL) were pelleted at 30,000 x *g* in a benchtop microcentrifuge and resuspended in solution 1 (100 µL) (50 mM Tris/HCl, pH 8; 10 mM EDTA). Alkaline lysis was performed by adding solution 2 (200 µL) (200 mM NaOH; 1% SDS) and mixing by inverting. Solution 3 (150 µL) (3 M potassium acetate, pH 5.5) was added and samples mixed by inverting, before the soluble material was harvested by microcentrifugation at 30,000 × *g* for 5 min. The nucleic acid was extracted with 25:24:1 phenol:chloroform:isoamyl alcohol (400 µL), and DNA was precipitated in 600 µL ice-cold isopropanol. After centrifugation, the resulting DNA pellet was washed in 200 µL 70% ethanol and air dried before being resuspended in water for quantification on a Nanodrop 2000c UV–Vis spectrophotometer. Plasmid DNA was isolated from *E. coli* strains using a Qiagen miniprep kit according to the manufacturer’s instructions. PAC pESAC13–215-G DNA, containing the entire *for* BGC, was used as the template for all PCR reactions. DNA amplification for cloning was conducted using Q5 Polymerase and diagnostic PCR was set up using PCRBIO Taw Mix Red (PCR Biosystems), as per the manufacturers’ instructions. Amplified fragments and digested DNA products were purified on 1% agarose gels by electrophoresis and extraction using the Qiagen gel extraction kit as per the manufacturer’s instructions. For overlay bioassays soft LB agar (100 mL LB with 0.5% agar) was inoculated with *Bacillus subtillis* 168 (10 mL; approximately OD_600 = _0.6). Set concentrations of the compound for testing were made up in methanol and an aliquot (20 µL) was applied to Whatman 6 mm antibiotic assay discs, air dried, and the discs placed in the centre of the solidified agar plates. Plates were incubated at 30 °C overnight before being examined for zones of inhibition around the discs. For liquid culture bioassays, cultures of *B. subtillis* 168 (1 mL) were grown at 30 °C, 200 rpm shaking, with the relevant antibiotic (50 µg mL^−1^). After 7 h incubation, samples were taken from the cultures, diluted in series and plates for colony count in triplicate following Miles and Misra protocol^34^.

### Generating mutant strains of *S. formicae*

CRISPR/Cas9 genome editing was conducted using the pCRISPomyces-2 plasmid supplied by Addgene. Protospacers to use in the synthetic guide RNA (sgRNA) were annealed by heating to 95 °C for 5 min followed by ramping to 4 °C at 0.1 °C s^−1^. Annealed protospacers were assembled into the pCRISPomyces-2 backbone at the *Bb*sI site by Golden Gate assembly. The two homology repair template arms (each ∼1 kb) were PCR-amplified as above and assembled into the plasmid containing the sgRNA at the *Xba*I site using Gibson Assembly^5,31^. Genetic complementation was achieved using either the native promoter or the constitutive, high-level ermE* promoter and single copies of the relevant gene(s) cloned into the integrative vector pMS82 or pIJ10257, respectively. Gibson assembly was used to fuse the gene(s) (and the native promoter if located distally in the BGC) and assemble them into the chosen plasmid. Plasmids were confirmed by PCR amplification and sequencing. Plasmids were then conjugated into *S. formicae* KY5 via the non-methylating *E. coli* strain ET12567 containing pUZ8002^35^. Ex-conjugants were selected on the appropriate antibiotics and pCRISPomyces-2 plasmids were cured from *S. formicae* using temperature selection at 37 °C.

### Isolation and structure determination of formicapyridines

*S. formicae* was cultivated on MS agar (14 L, ∼450 plates) at 30 °C for 9 days. The agar was sliced into small pieces and extracted twice with ethyl acetate (10 L) using ultrasonication to improve the extraction. The extracts were combined, and the solvent removed under reduced pressure to yield a brown oil, which was dissolved in methanol (20 mL). This extract was first chromatographed over a Phenomenex Gemini-NX reversed-phase column (C_18_, 110 Å, 150 × 21.2 mm) using a Thermo Scientific Dionex Ultimate 3000 HPLC system and eluting with the following gradient method: (mobile phase A: water +0.1% formic acid; mobile phase B: acetonitrile) 0–5 min 40% B; 5–35 min 40–100% B; 35–40 min 100% B; 40–40.1 min 100–40% B; and 40.1–45 min 40% B; flowrate 20 mL min^−1^; injection volume 1 mL. Absorbance was monitored at 250 nm. Fractions (20 mL) were collected and analysed by LCMS. Fractions 2–4 contained **1**–**6** and were further purified by chromatography over a Phenomenex Gemini-NX semi-prep reversed-phase column (C_18_, 110 Å, 150 × 10 mm) using an Agilent 1100 series HPLC system and eluting with the following gradient method: (mobile phase A: water +0.1% formic acid; mobile phase B: acetonitrile) 0–2 min 40% B; 2–20 min 40–100% B; 20–21 min 100% B; 21–21.1 min 100–40% B; 21.1–23 min 40% B; flowrate 3 mL min^−1^; injection volume 100 µL). Absorbance was monitored at 390 nm. The samples were finally purified by Sephadex LH20 size exclusion chromatography with 100% methanol as the mobile phase. The isolated yields were: **1** (1 mg), **2** (2 mg), **3** (2 mg), **4** (0.7 mg), **5** (1 mg) and **6** (0.6 mg). These pure compounds were subjected to analysis by HRMS and 1D and 2D NMR as described in the main text (see Figs. 1 and 2). Spectroscopic and other data for each compound is presented in Supplementary Information (Supplementary Figs. 15–38 and Supplementary Note 1).

### Stable isotope feeding experiment

*S. formicae* was cultivated on MS agar (3 L; ∼100 plates) at 30 °C and overlaid with [1,2-^13^C_2_] sodium acetate (1 mL of a 60 mM solution) after 24 h, 48 h, 72 h, 96 h and 120 h. After a further 72 h the agar was extracted and purified using the methods described above to yield a sample of **2** (0.9 mg). This material was analyzed by LCMS and inverse gated ^13^C NMR (125 MHz; 4096 scans; *d*_4_-methanol). However, due to the weak and overlapping signals, only the following coupling constants (*J*_CC_) of the intact acetate units were recorded: C24–C1, 44.61 Hz; C2–C3, 68.58 Hz; C4–C5, 68.16 Hz; C20–C21, 56.18 Hz. In addition, C14, C16, C18 and C22 have coupling constants of 66.47, 67.61, 42.08 and 60.43 Hz, respectively. Spectroscopic data is presented in Fig. 5 and Supplementary Fig. 4.

### Isolation and structure determination of fasamycin F (13)

*S. formicae ΔforS* was cultivated on 4 L (~120 plates) of MS agar at 30 °C for nine days. The agar was extracted and purified using the methods described above to yield **13** (3.4 mg). This material was analysed by LCMS and 1D and 2D NMR (100 MHz; 6500 scans; *d*_4_-methanol). Spectroscopic data is presented in Supplementary Figs. 39–43.

### Congener content analysis of cyclase mutants

*S. formicae* WT or mutant strains (*n* = 3) were grown on MS agar at 30 °C for nine days. A rectangle of agar (2 cm^3^) was excised from each petri dish, sliced into small pieces and shaken with ethyl acetate (1 mL) for 20 min. The ethyl acetate was transferred to a clean tube and the solvent removed under reduced pressure. The resulting extract was dissolved in methanol (200 µL) and analysed by LCMS but using the following modified UPLC method: Phenomenex Gemini C18 column (100 × 2.1 mm, 100 Å); mobile phase A: water +0.1% formic acid; mobile phase B: methanol. Elution gradient: 0–2 min, 50% B; 2–14 min, 50–100% B; 14–18 min, 100% B; 18–18.1 min, 100–50% B; 18.1–20 min, 50% B; flow rate 1 mL min^−1^; injection volume 10 μL.

Calibration curves (Supplementary Fig. 10; Supplementary Data 3) were determined using standard solutions of fasamycin C **10** (10, 20, 50, 80 and 200 µM), formicamycin C **16** (10, 20, 50, 100 and 200 µM), formicapyridine D **4** (5, 10, 25, 50 and 100 µM) and fasamycin F **13** (5, 20, 40, 80 and 150 µM). The content of **10** and **16** was determined by UV absorption at 285 nm. The content of **4** and **13** was determined by MS analysis of the base peak chromatogram (positive mode). Each standard solution was measured three times.

### Reporting summary

Further information on research design is available in the Nature Research Reporting Summary linked to this article.

## Supplementary information

Supplementary Information

Peer Review File

Reporting Summary

Description of Additional Supplementary Files

Supplementary Data 1

Supplementary Data 2

Supplementary Data 3

Supplementary Data 4

Supplementary Data 5

Source Data

## Data Availability

Data supporting the findings of this work are available within the paper and its Supplementary Information files. A reporting summary for this Article is available as a Supplementary Information file. The datasets generated and analyzed during the current study are available from the corresponding author upon request. The source data underlying Fig. 6e–h are provided as a Source Data file.
