# Supplementary material for: A role for antibiotic biosynthesis monooxygenase domain proteins in fidelity control during aromatic polyketide biosynthesis
Source: Nat Commun. 2019 Aug 9;10:3611. doi: 10.1038/s41467-019-11538-6 (PMC6689052; doi:10.1038/s41467-019-11538-6)
Supplement: Supplementary file 1 — Supplementary Information [file 41467_2019_11538_MOESM1_ESM.pdf]

A role for antibiotic biosynthesis monooxygenase domain proteins in fidelity control during aromatic polyketide biosynthesis

Qin *et al.*

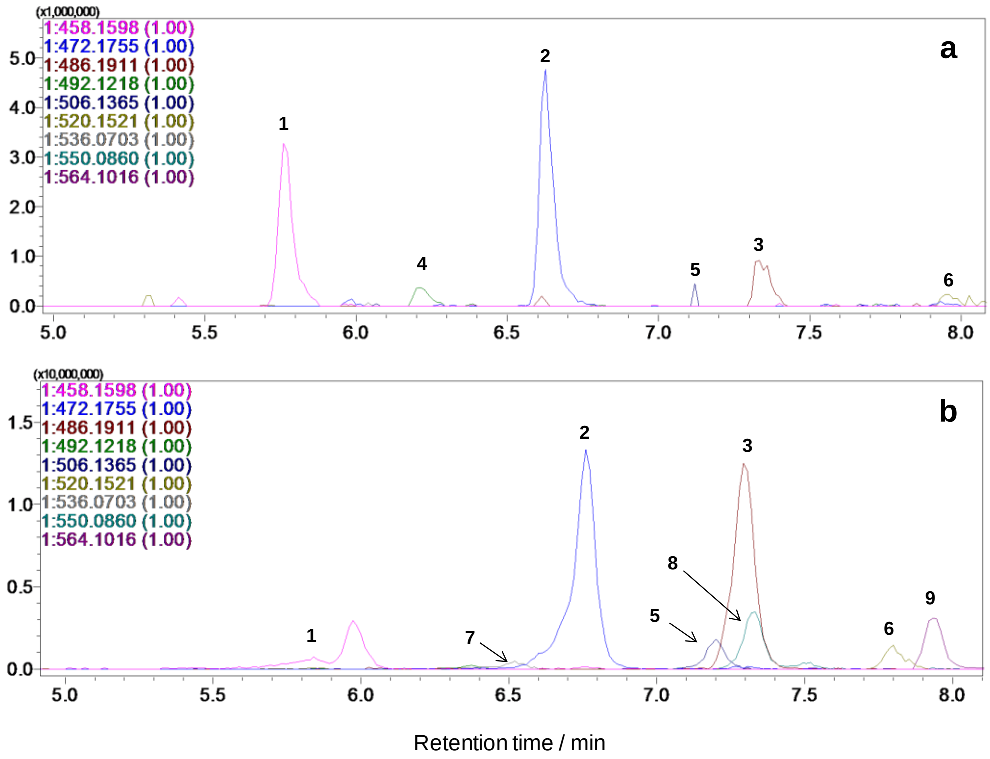

Supplementary Figure 1. Extracted ion chromatograms for 1-9 from LCMS runs of extracts from the WT strain grown on MS agar medium. (a) without exogenous addition of NaBr; (b) with exogenous addition of NaBr. The slight shifts in retention times for **1**-**6** was due to machine maintenance carried out between the experiments.

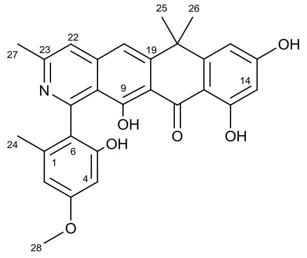

Formicapyridine B (**2**)

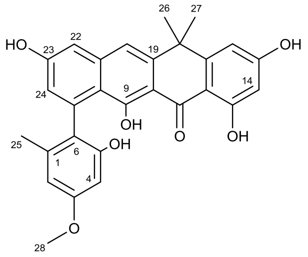

Fasamycin C (**10**)

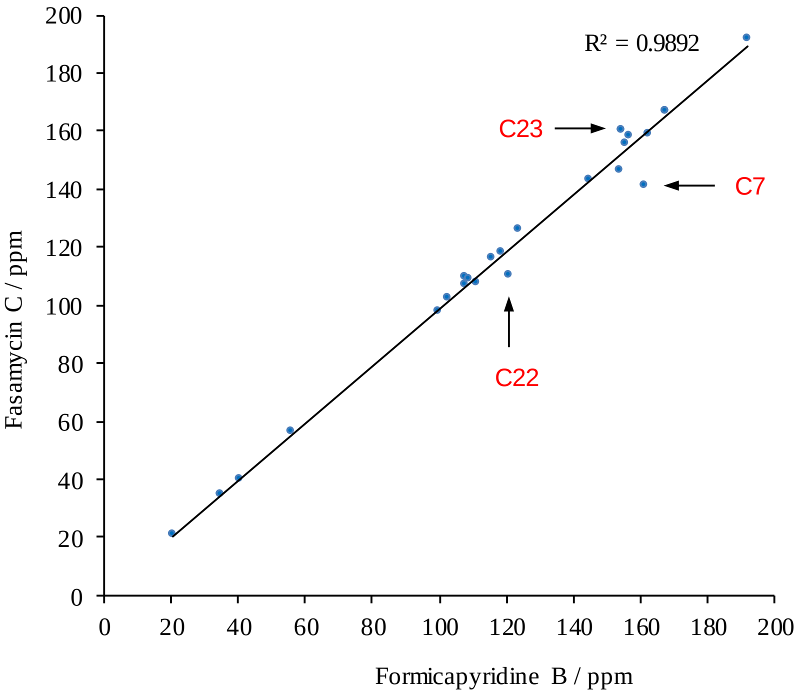

Supplementary Figure 2. Comparison of the ^{13}C NMR chemical shifts for formicapyridine B and fasamycin C. The chemical shifts for carbons C7, C22 and C23 are highlighted.

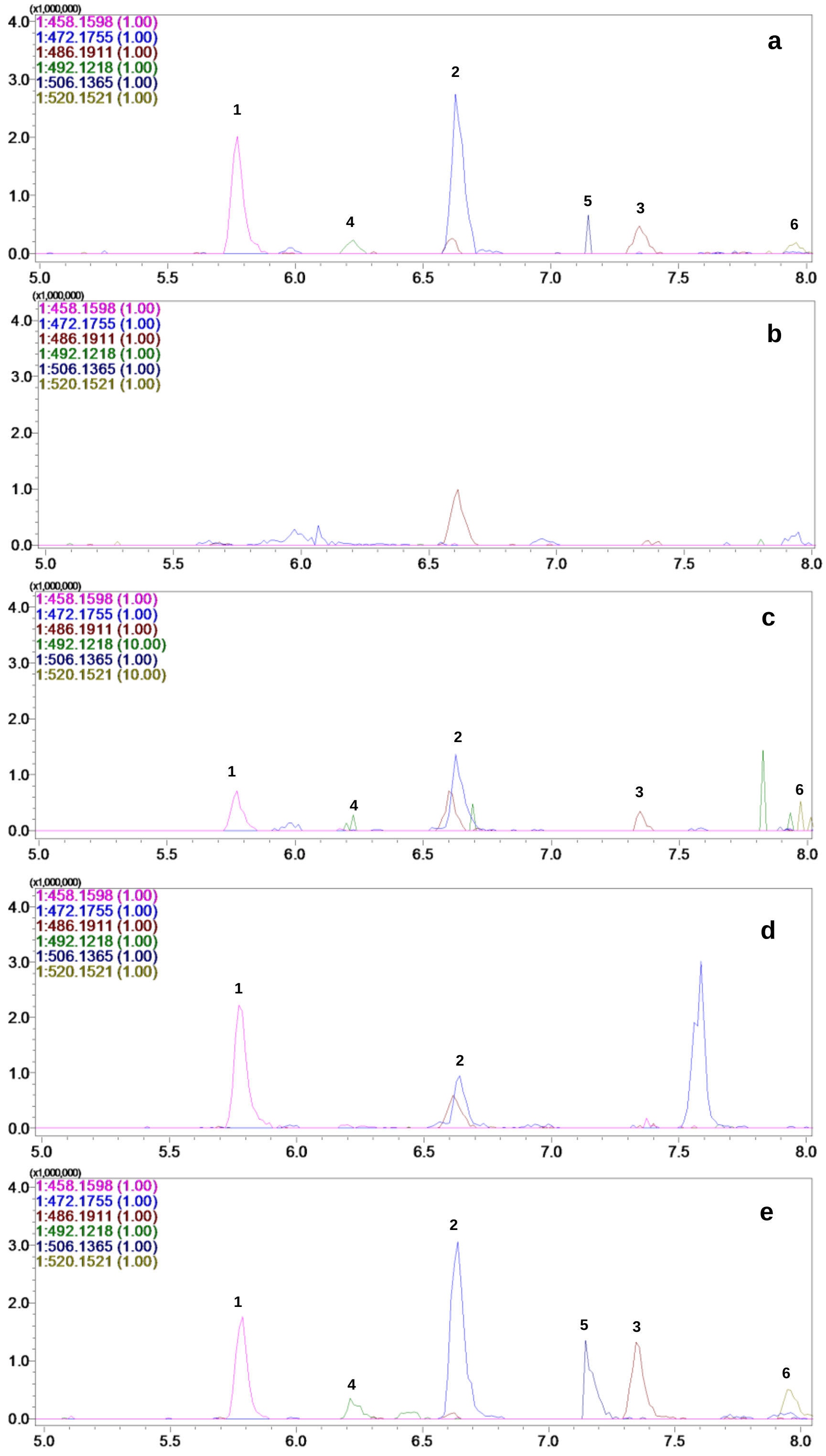

Supplementary Figure 3. Reconstituted LCMS extracted ion chromatograms for 1-6. (a) *S. formicae* WT extract; (b) *S. formicae* Δ*for* extract; (c) *S. formicae* Δ*for/for* extract; (d) *S. formicae* Δ*forV* extract; (e) *S. formicae* Δ*forV/forV* extract. The signal abundance of **4** and **6** in (c) was enlarged ×10 for clarity.

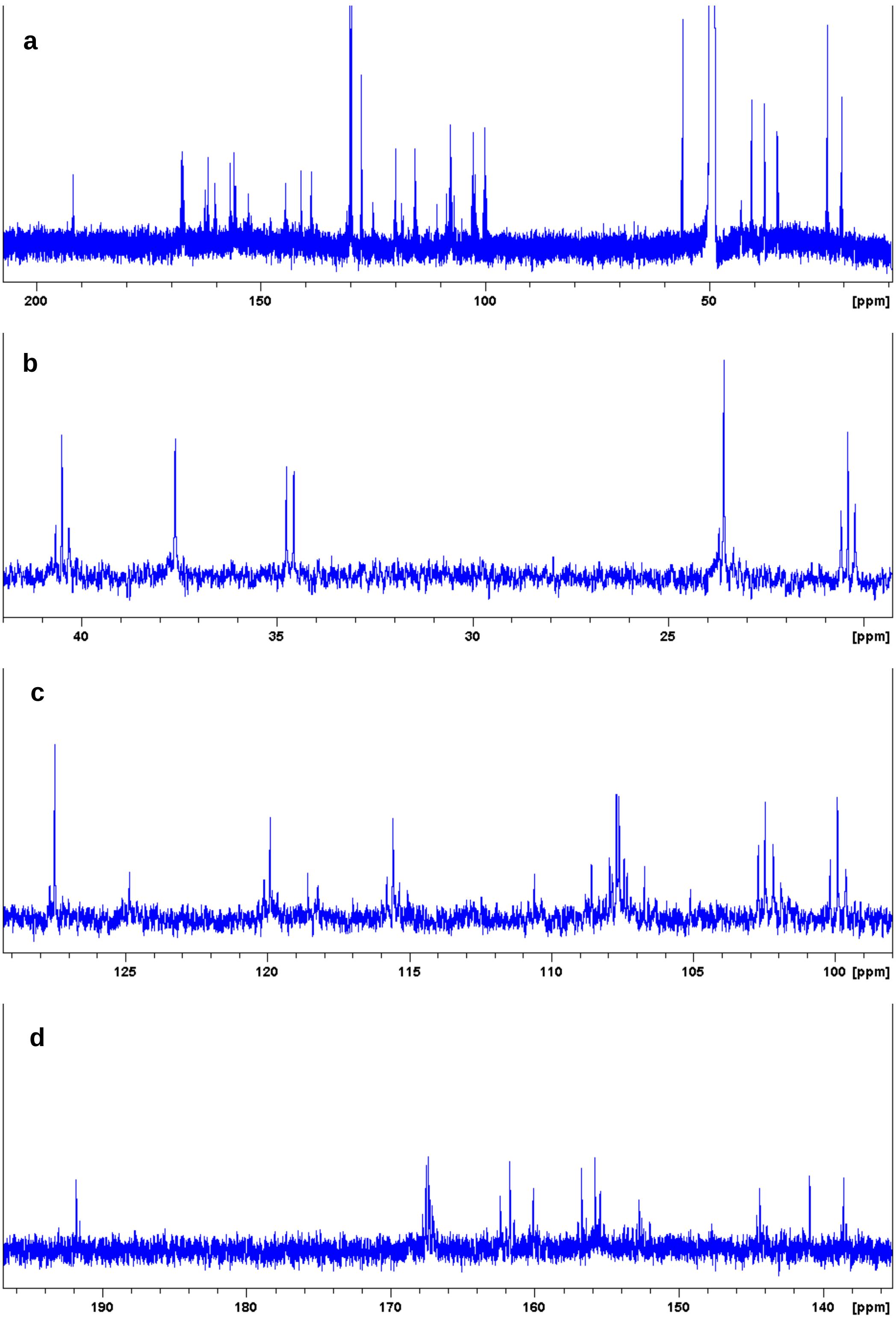

Supplementary Figure 4. ^{13}C NMR spectra of 2 isolated after growth of *S. formicae* WT in the presence of [1,2-$^{13}C_2$] sodium acetate. (a), full scale; (b)-(d), expanded scale. Condition: 125 MHz, methanol-d_6.

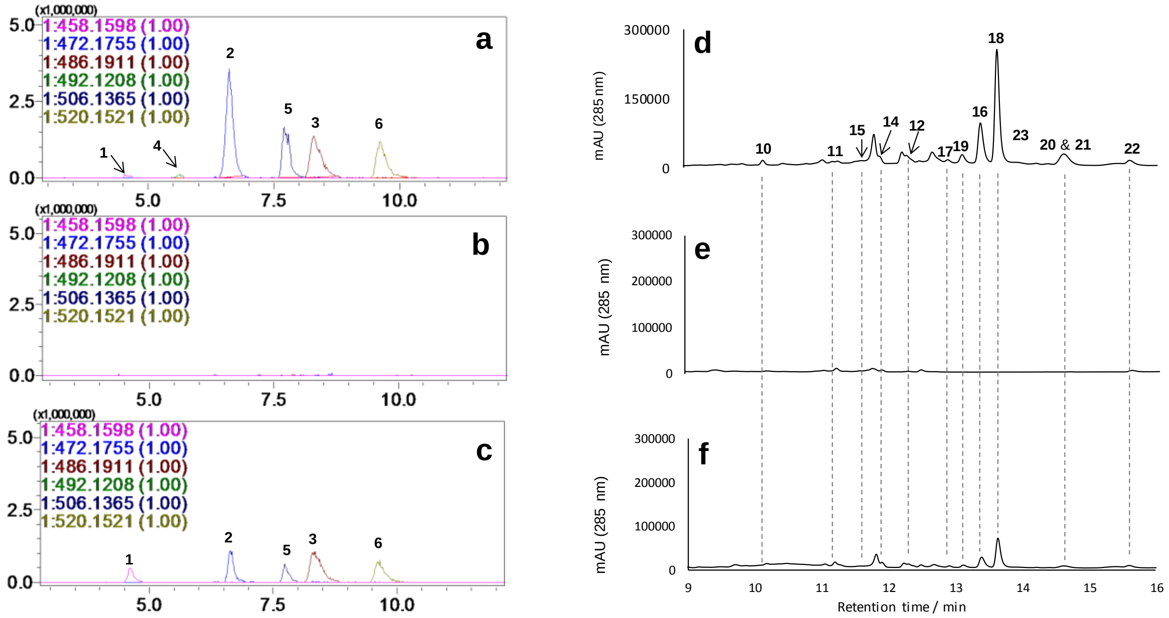

Supplementary Figure 5. Mutational analysis of *forD*. Extracted ion chromatogram (left) and reconstituted HPLC-UV (285 nm) profiles (right) for extracts of *forD* mutagenesis and complementation experiments: (a) and (d) *S. formicae* wild-type; (b) and (e) *S. formicae* Δ*forD*; (c) and (f) *S. formicae* Δ*forD/forD*.

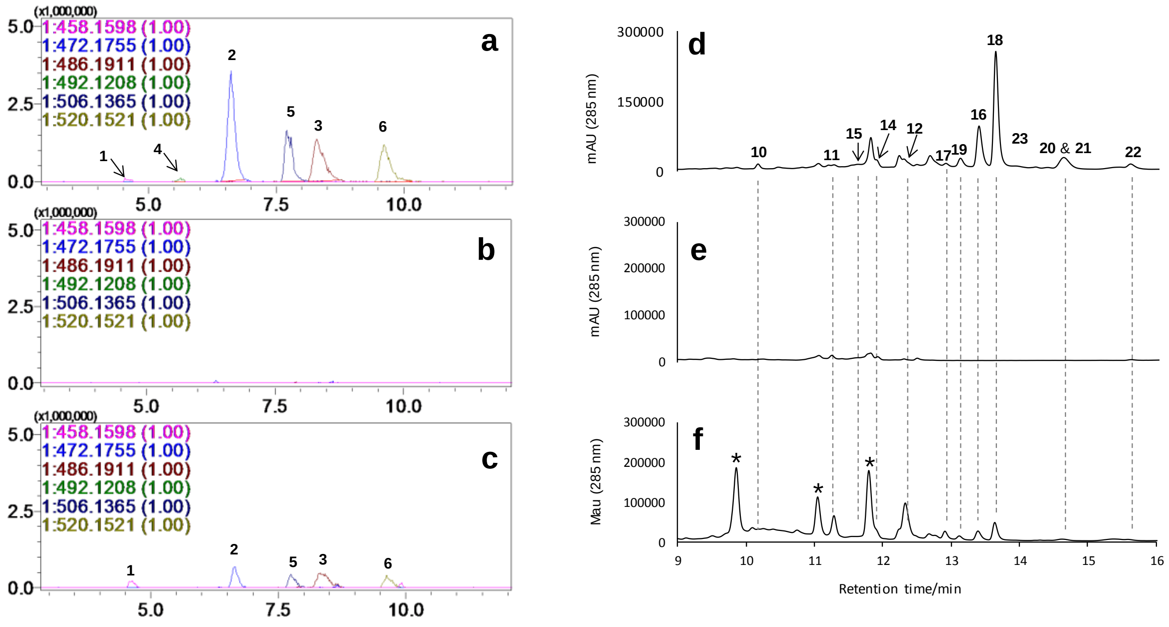

Supplementary Figure 6. Mutational analysis of *forL*. Extracted ion chromatogram (left) and reconstituted HPLC-UV (285 nm) profiles (right) for extracts of *forL* mutagenesis and complementation experiments: (a) and (d) *S. formicae* wild-type; (b) and (e) *S. formicae* Δ*forL*; (c) and (f) *S. formicae* Δ*forL/forL*. The molecular species giving rise to peaks labelled with an asterisk * are unrelated to the *for* pathway and correspond to a metabolite produced variably by *S. formicae* KY5.

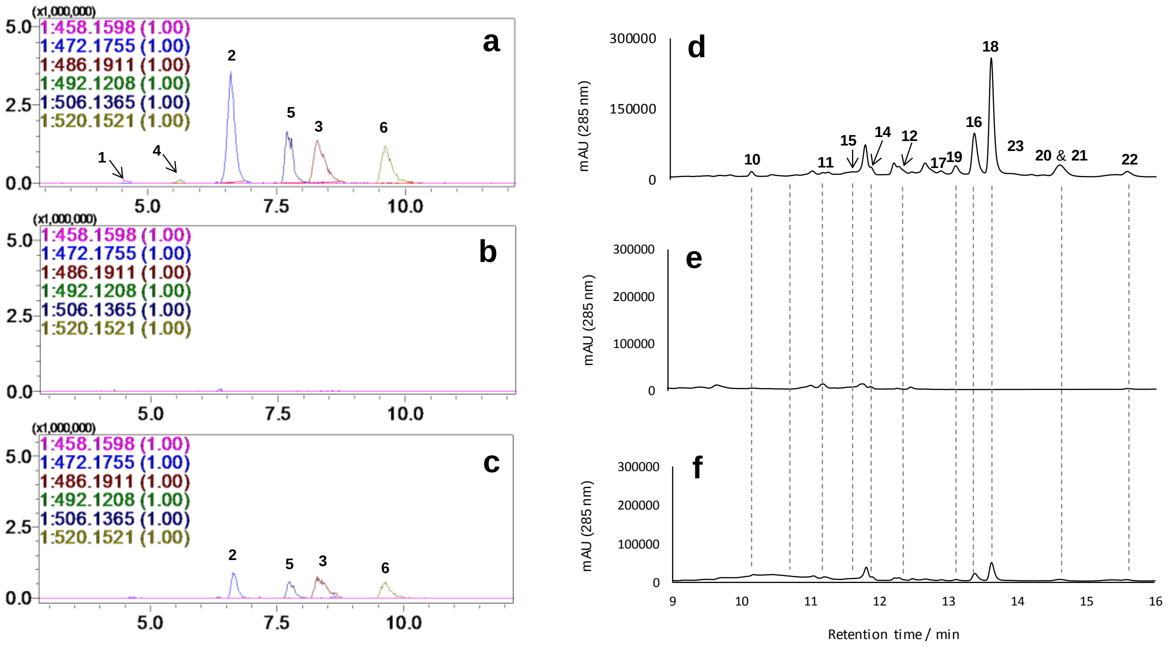

Supplementary Figure 7. Mutational analysis of *forR*. Extracted ion chromatogram (left) and reconstituted HPLC-UV (285 nm) profiles (right) for extracts of *forR* mutagenesis and complementation experiments: (a) and (d) *S. formicae* wild-type; (b) and (e) *S. formicae* Δ*forR*; (c) and (f) *S. formicae* Δ*forR/forR*.

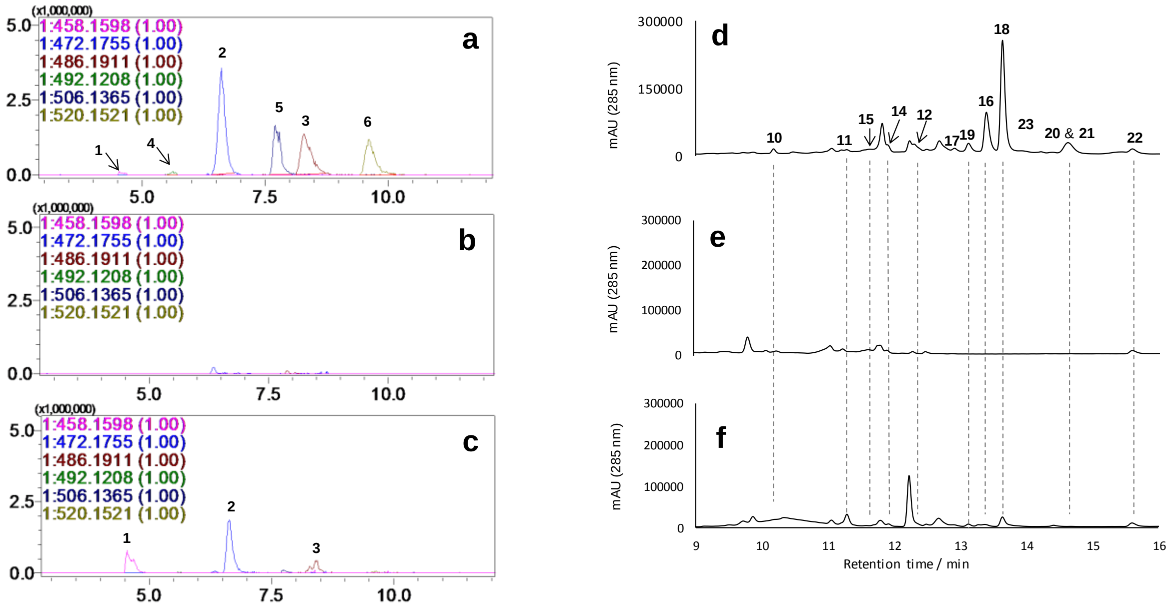

Supplementary Figure 8. Mutational analysis of *forU*. Extracted ion chromatogram (left) and reconstituted HPLC-UV (285 nm) profiles (right) for extracts of *forU* mutagenesis and complementation experiments: (a) and (d) *S. formicae* wild-type; (b) and (e) *S. formicae* Δ*forU*; (c) and (f) *S. formicae* Δ*forU/forUV*.

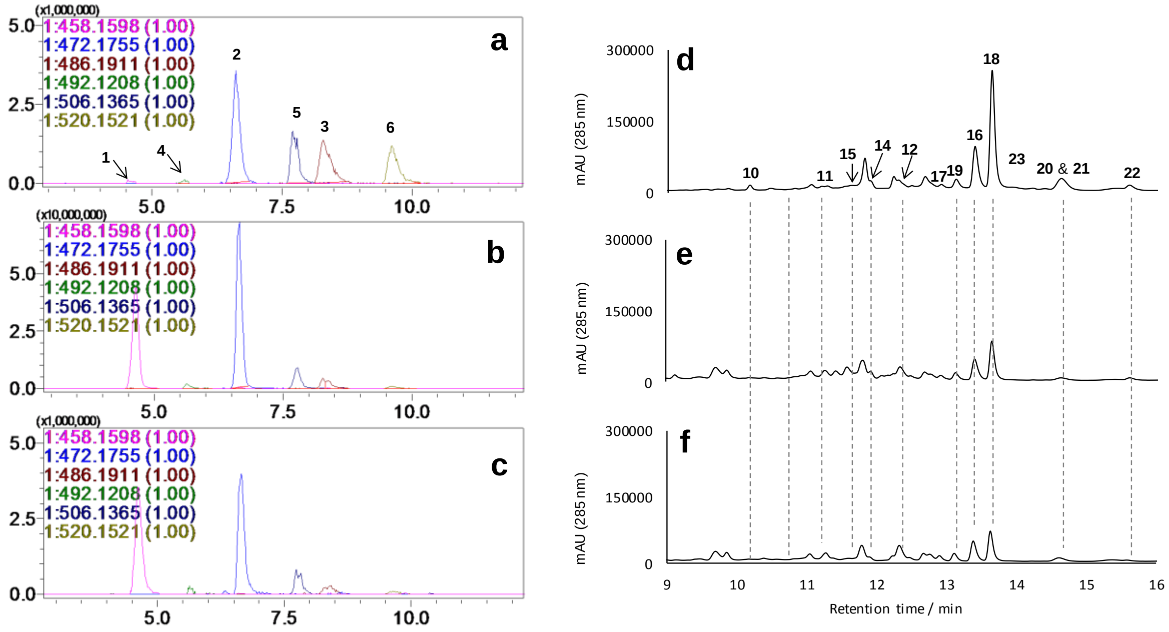

Supplementary Figure 9. Mutational analysis of *forS*. Extracted ion chromatogram (left) and reconstituted HPLC-UV (285 nm) profiles (right) for extracts of *forS* mutagenesis and complementation experiments: (a) and (d) *S. formicae* wild-type; (b) and (e) *S. formicae* Δ*forS*; (c) and (f) *S. formicae* Δ*forS/forS*.

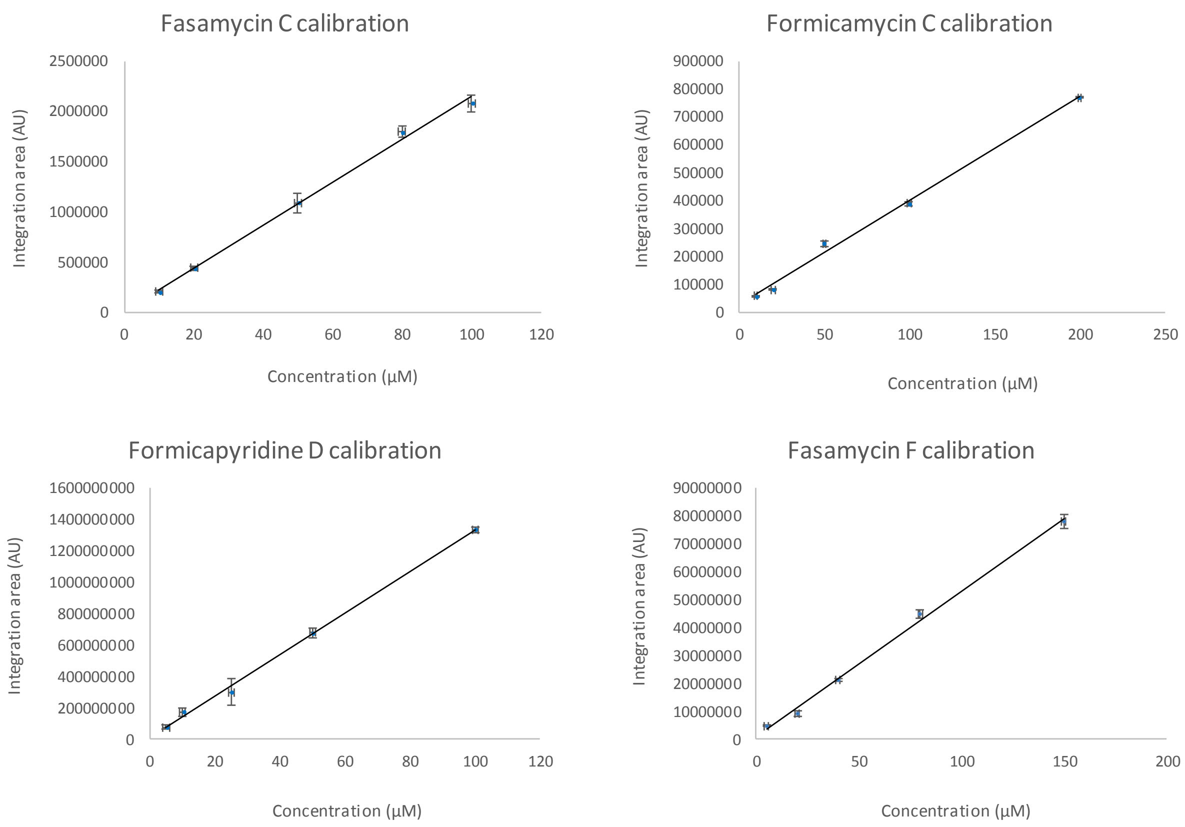

Supplementary Figure 10. Representative calibration curves. Ffasamycin C (**10**); formicamycin C (**16**); formicapyridine D (**4**); and fasamycin F (**13**). Each data point of the calibration curves is the mean ± standard deviation, *n* =3.

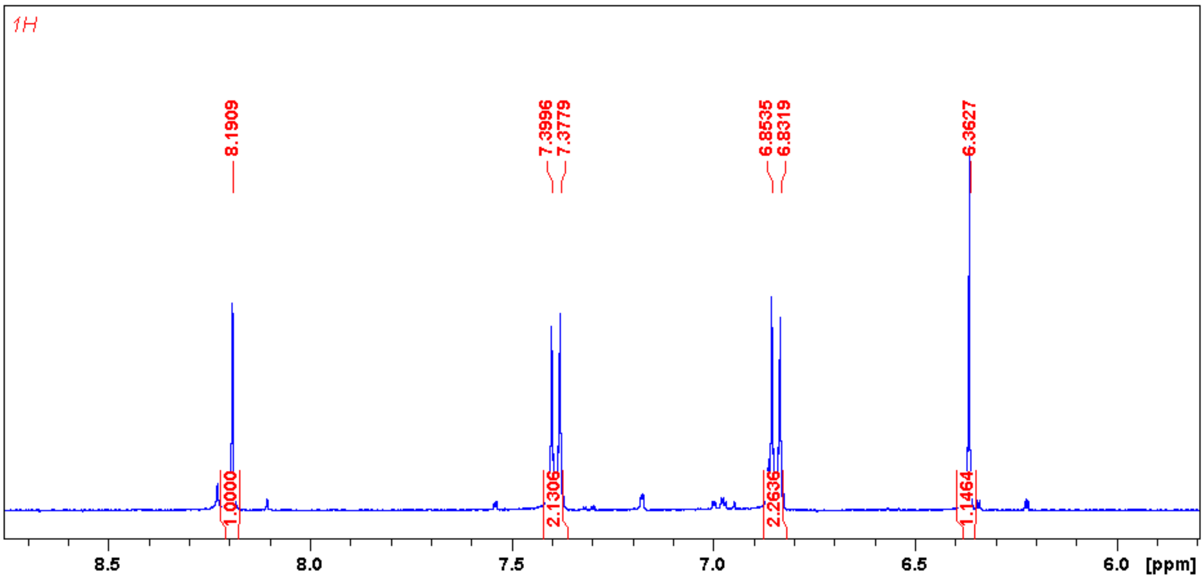

Supplementary Figure 11. ^{1}H NMR spectrum for 6-chlorogenistein. Condition: CD_3OD, 400 MHz.

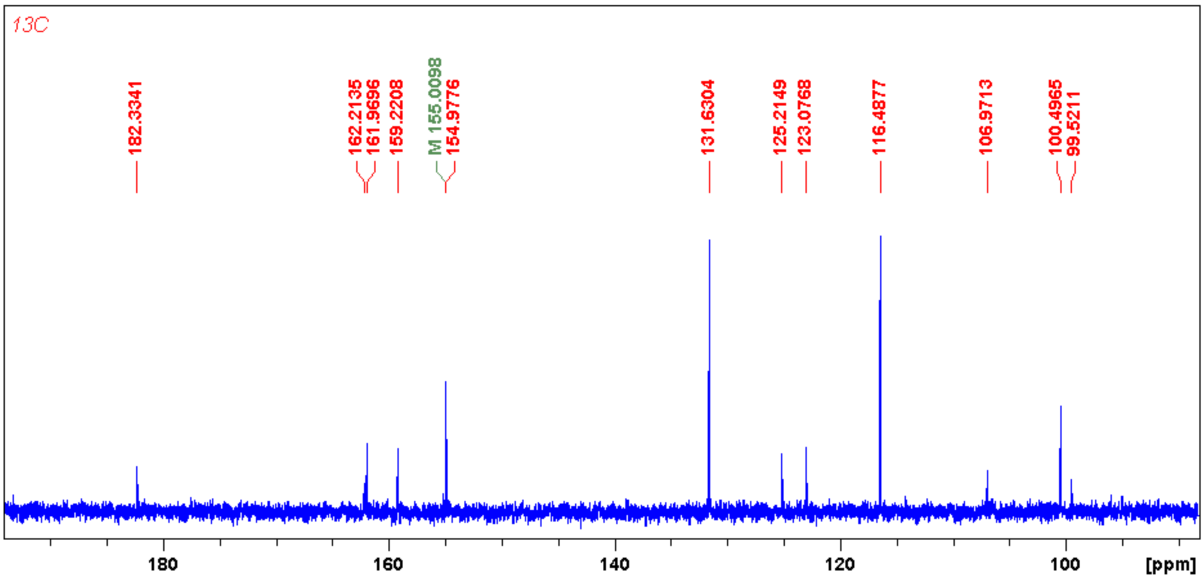

Supplementary Figure 12. ^{13}C NMR spectrum for 6-chlorogenistein. Condition: CD_3OD, 100 MHz.

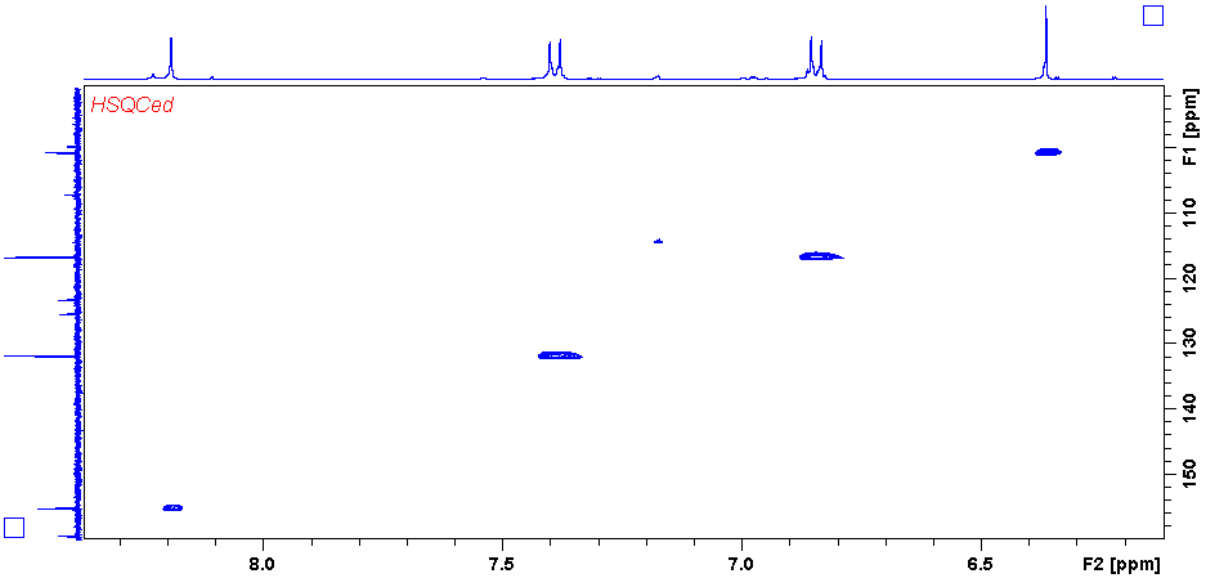

Supplementary Figure 13. HSQC spectrum for 6-chlorogenistein. Condition: CD_3OD.

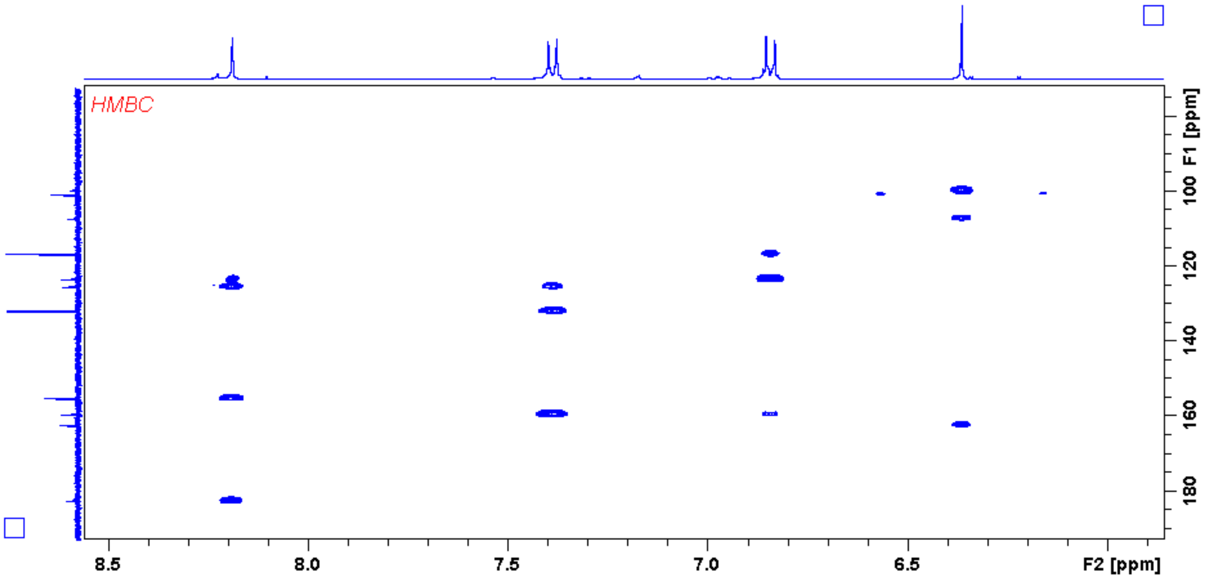

Supplementary Figure 14. HMBC spectrum for 6-chlorogenistein. Condition: CD_3OD.

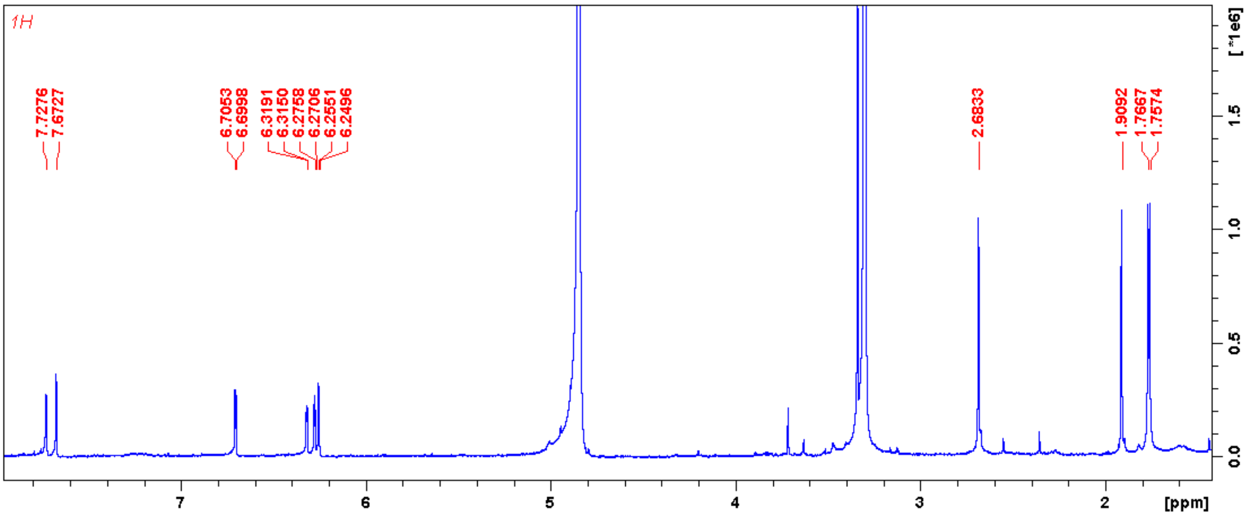

Supplementary Figure 15. ^{1}H NMR spectrum for compound 1. Condition: CD_3OD, 400 MHz.

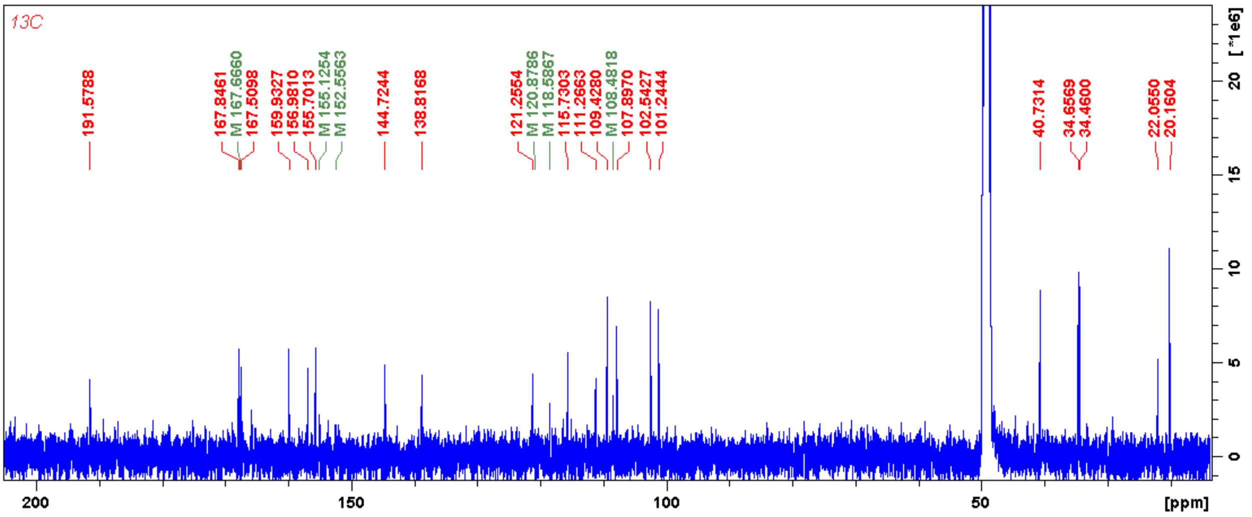

Supplementary Figure 16. ^{13}C NMR spectrum for compound 1. Condition: CD_3OD, 100 MHz.

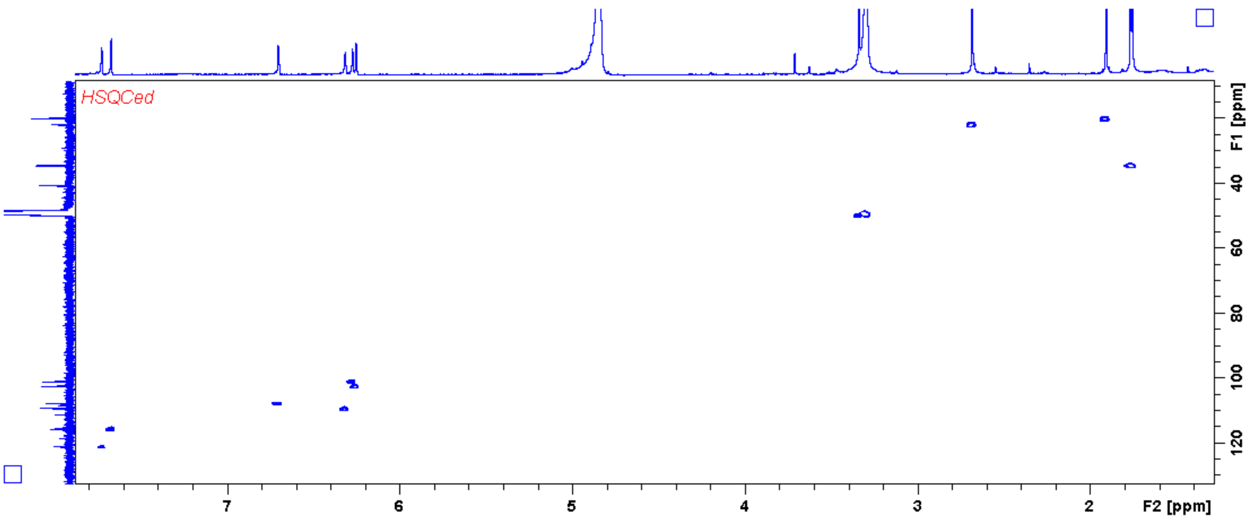

Supplementary Figure 17. HSQC spectrum for compound 1. Condition: CD_3OD.

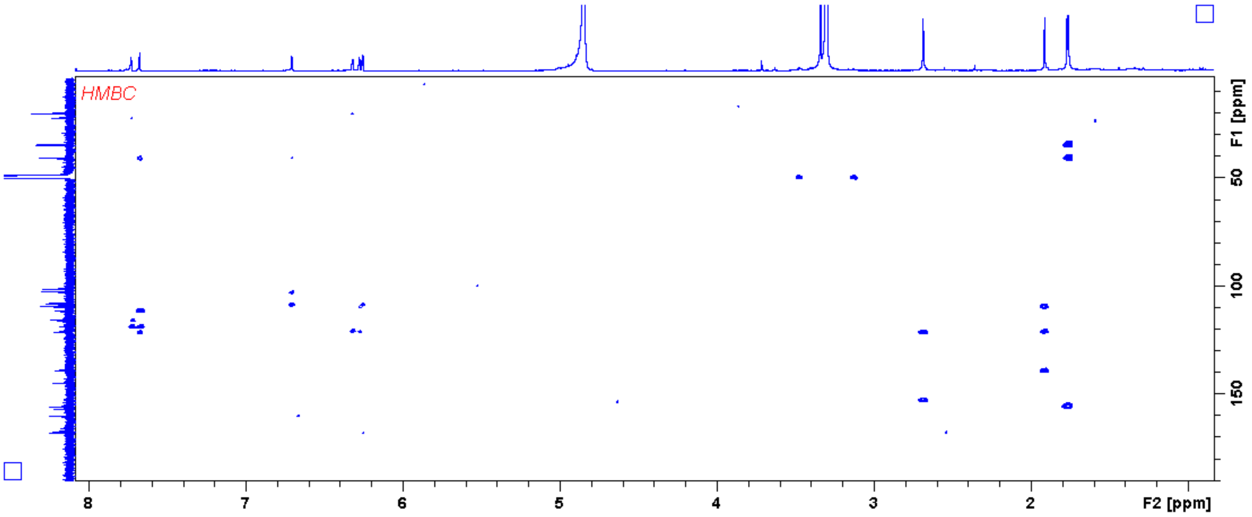

Supplementary Figure 18. HMBC spectrum for compound 1. Condition: CD_3OD.

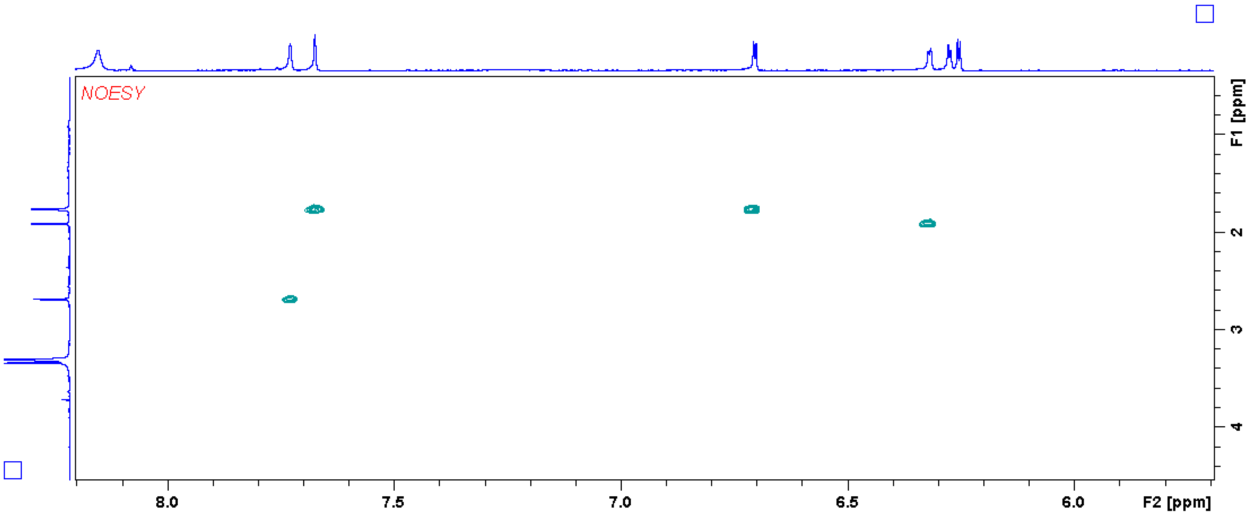

Supplementary Figure 19. NOESY spectrum for compound 1. Condition: CD_3OD.

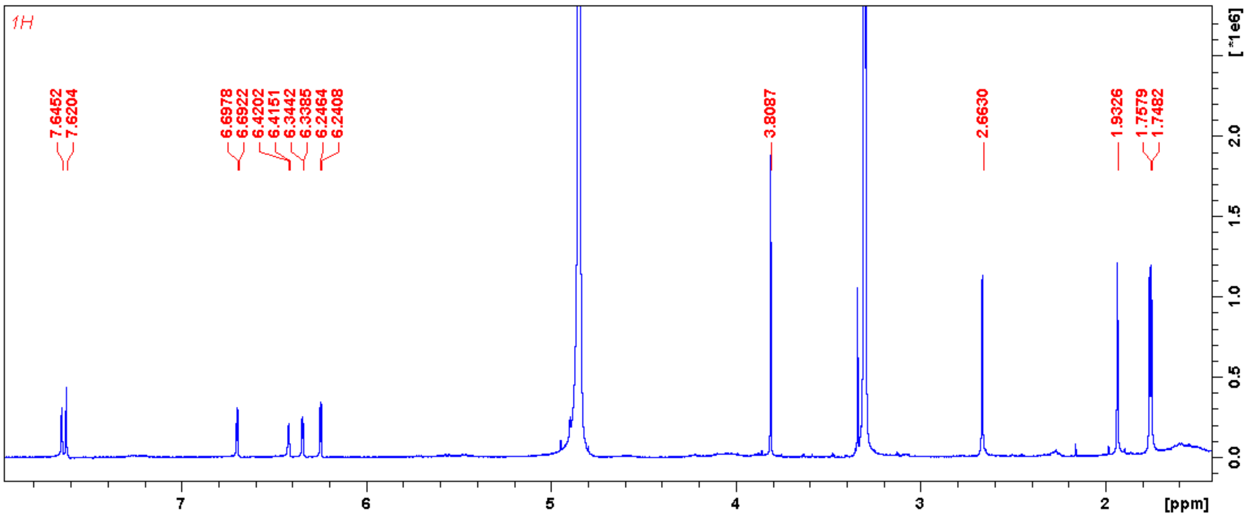

Supplementary Figure 20. ^{1}H NMR spectrum for compound 2. Condition: CD_3OD, 400 MHz.

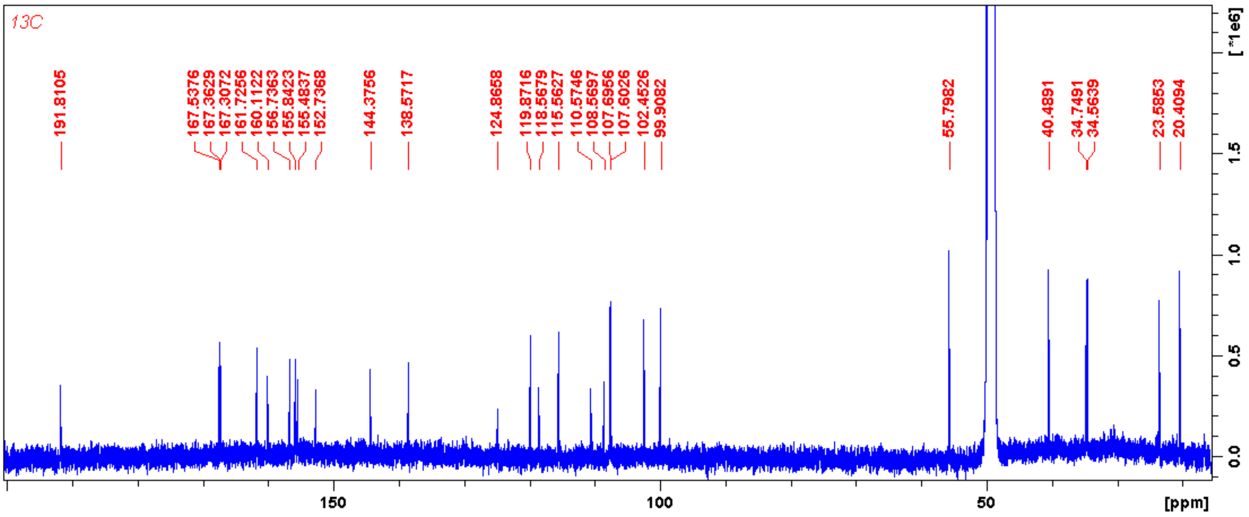

Supplementary Figure 21. ^{13}C NMR spectrum for compound 2. Condition: CD_3OD, 100 MHz.

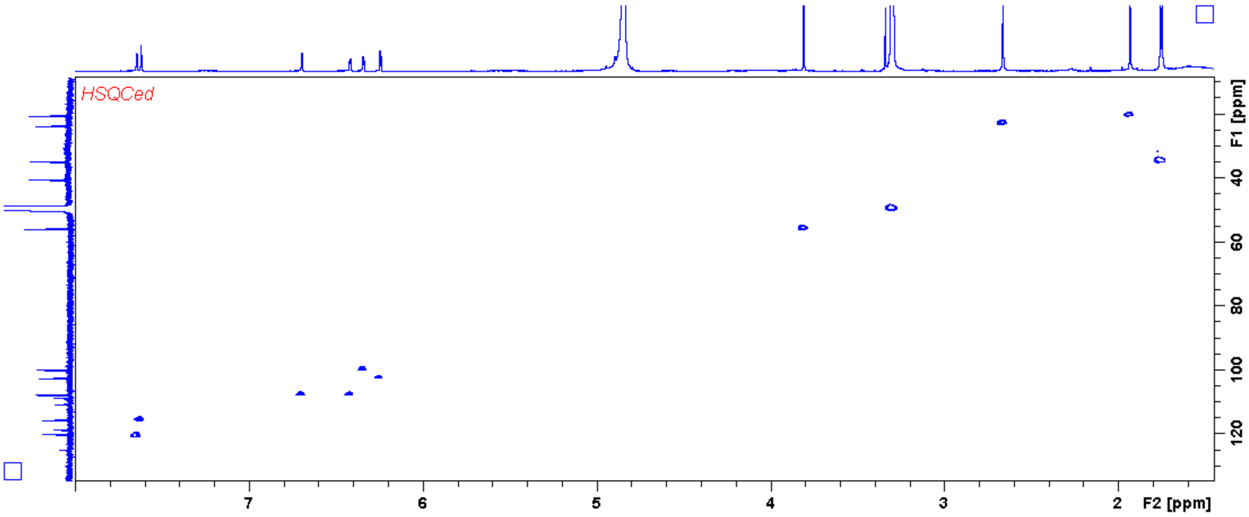

Supplementary Figure 22. HSQC spectrum for compound 2. Condition: CD_3OD.

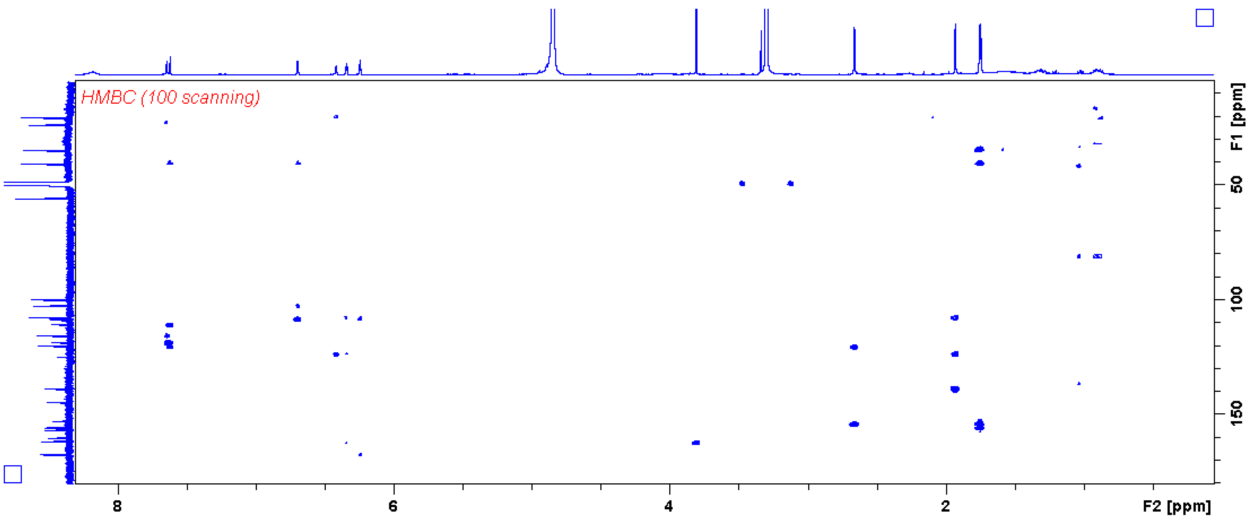

Supplementary Figure 23. HMBC spectrum for compound 2. Condition: CD_3OD.

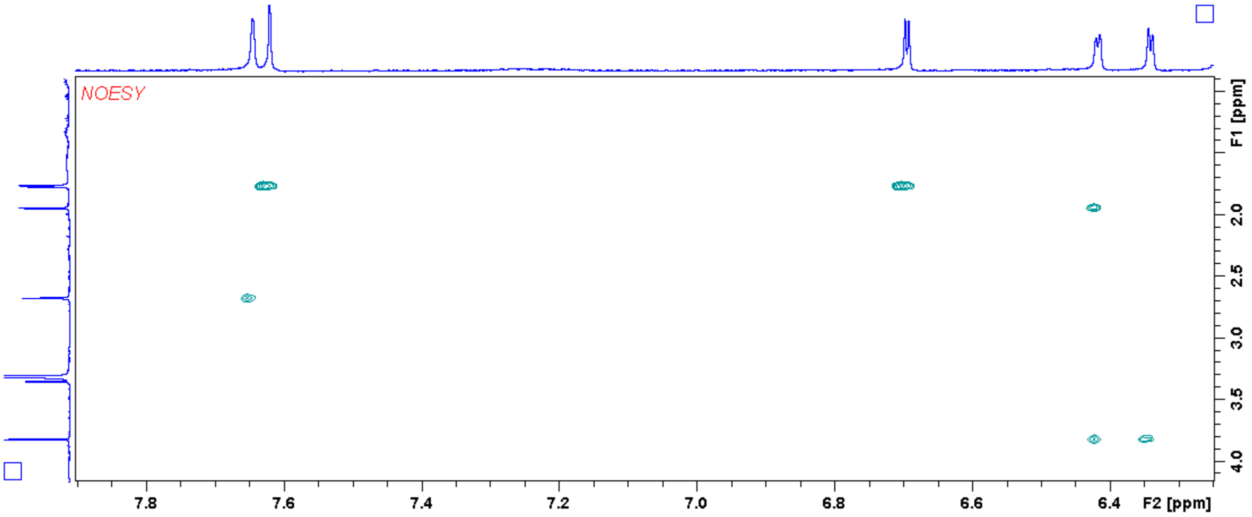

Supplementary Figure 24. NOESY spectrum for compound 2. Condition: CD_3OD.

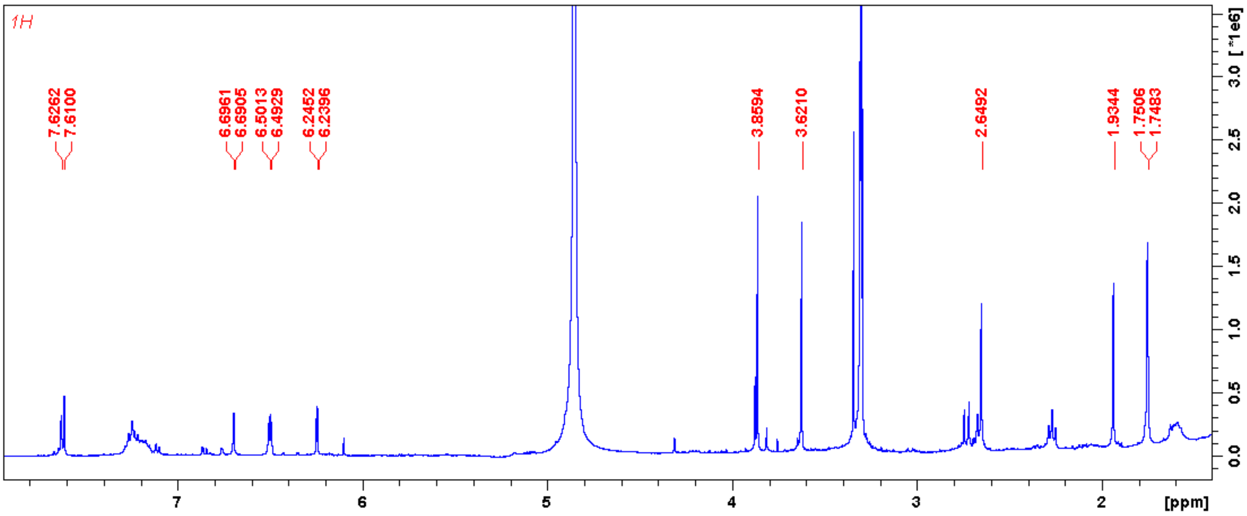

Supplementary Figure 25. ¹H NMR spectrum for compound 3. Condition: CD_3OD, 400 MHz.

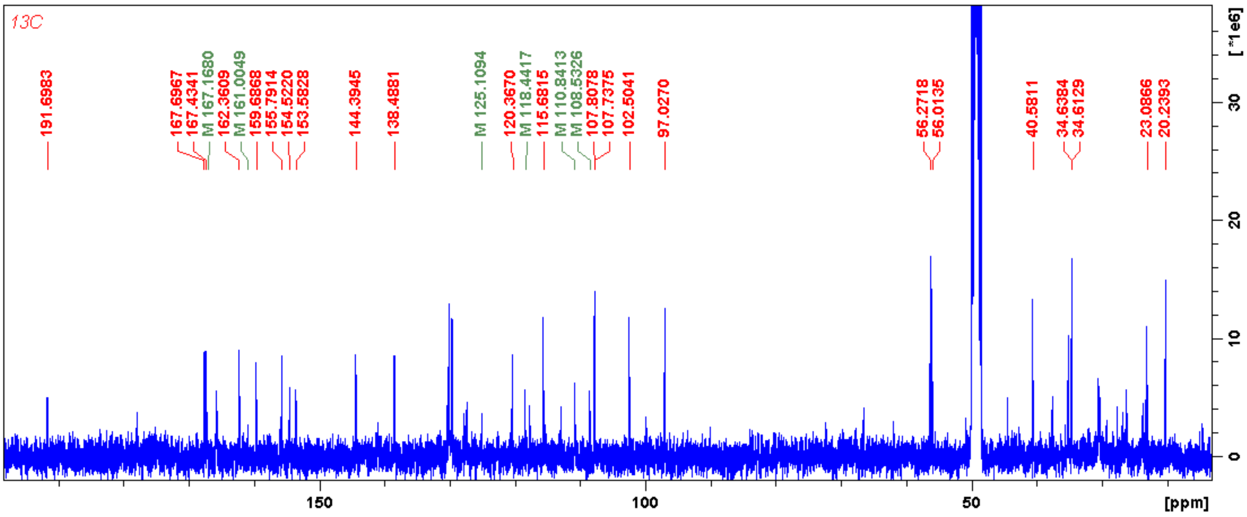

Supplementary Figure 26. ¹³C NMR spectrum for compound 3. Condition: CD_3OD, 100 MHz.

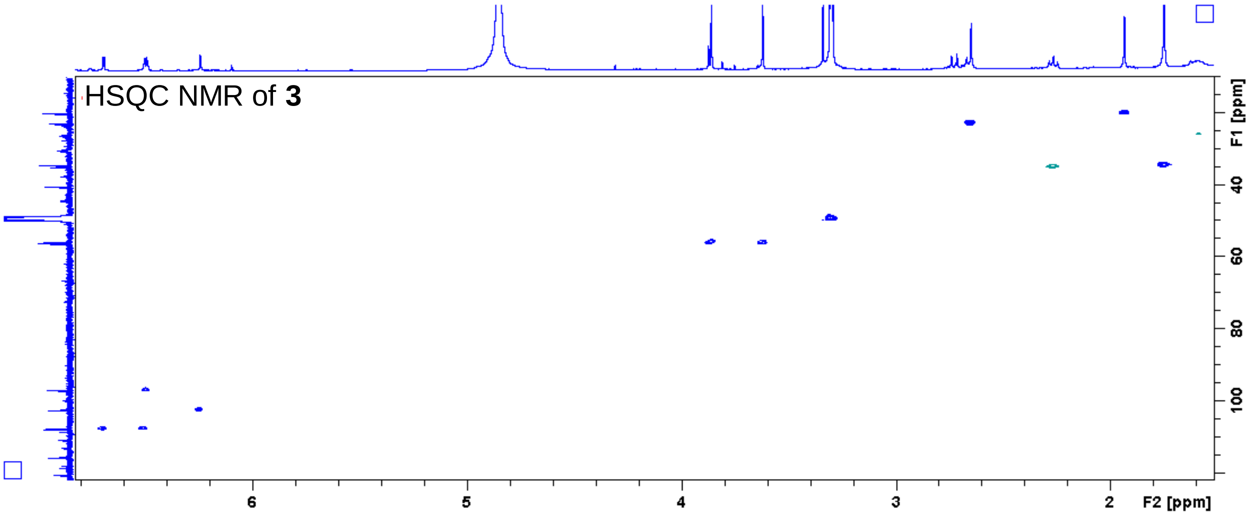

Supplementary Figure 27. HSQC spectrum for compound 3. Condition: CD_3OD.

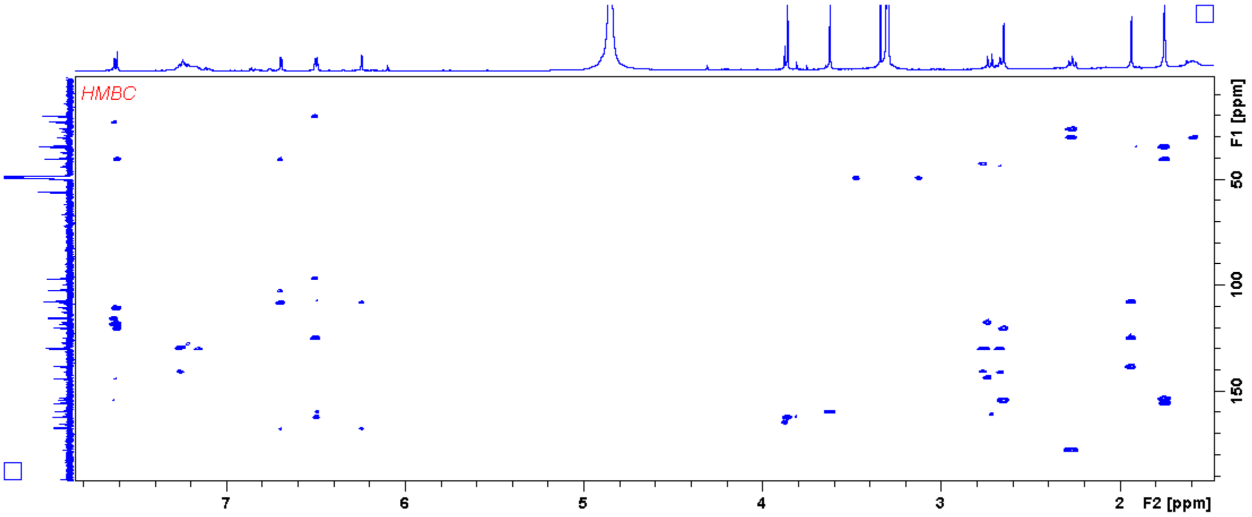

Supplementary Figure 28. HMBC spectrum for compound 3. Condition: CD_3OD.

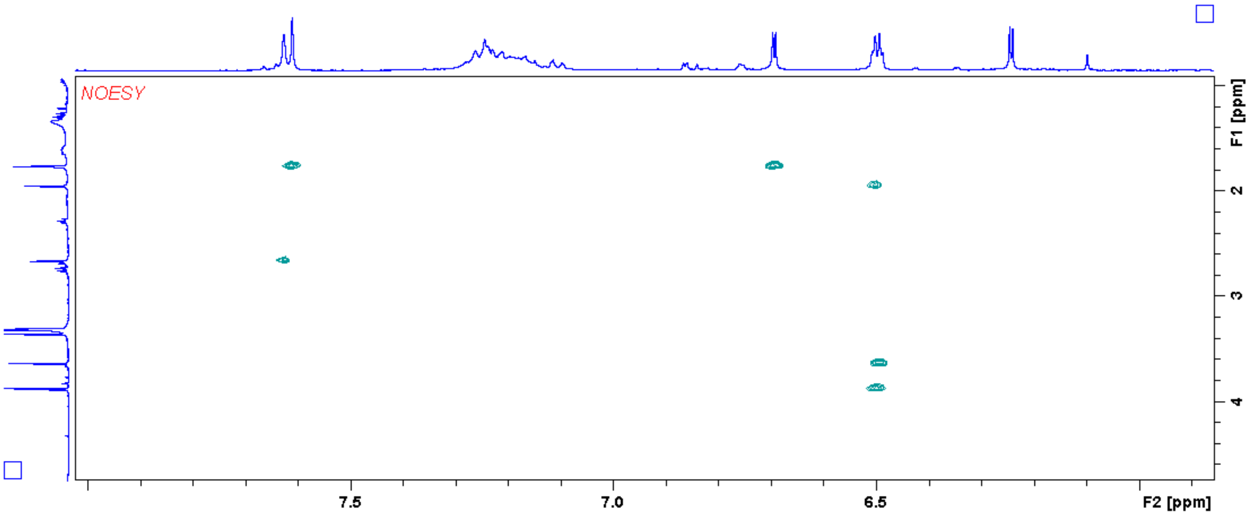

Supplementary Figure 29. NOESY spectrum for compound 3. Condition: CD_3OD.

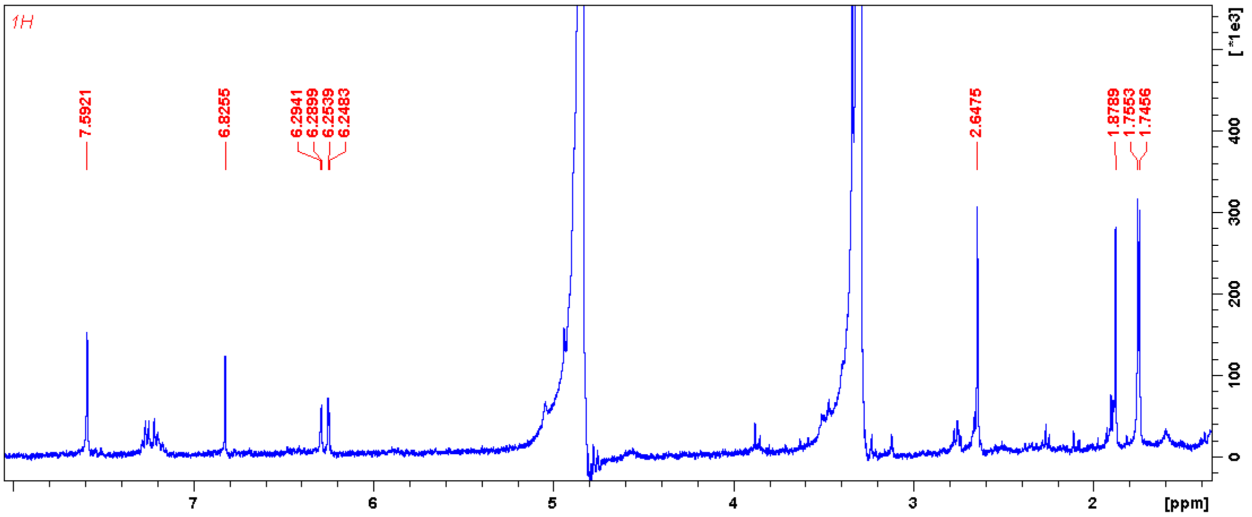

Supplementary Figure 30. ^{1}H NMR spectrum for compound 4. Condition: CD_3OD, 400 MHz.

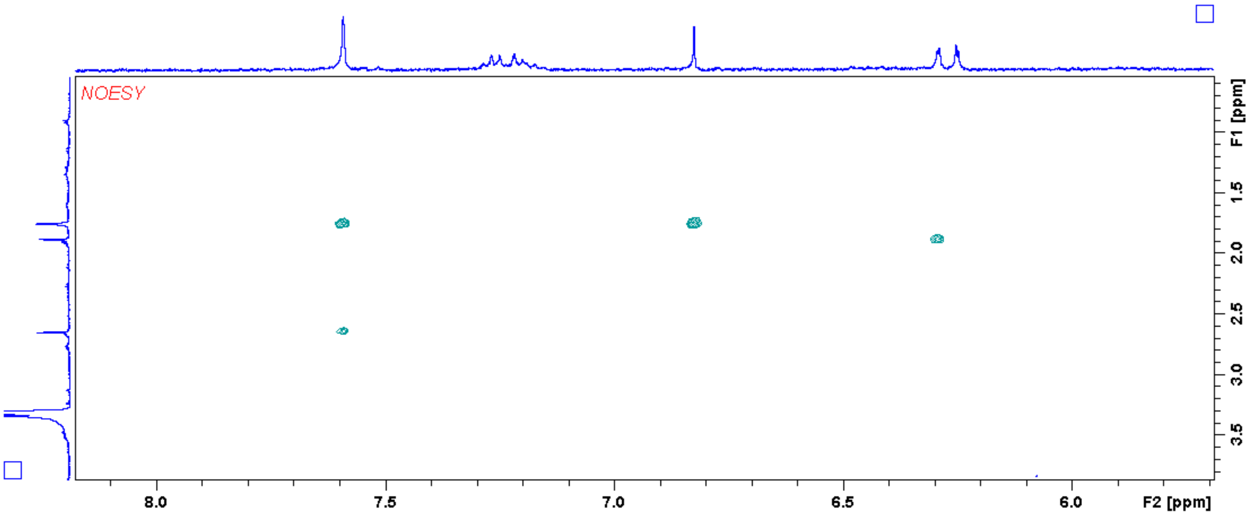

Supplementary Figure 31. NOESY spectrum for compound 4. Condition: CD_3OD.

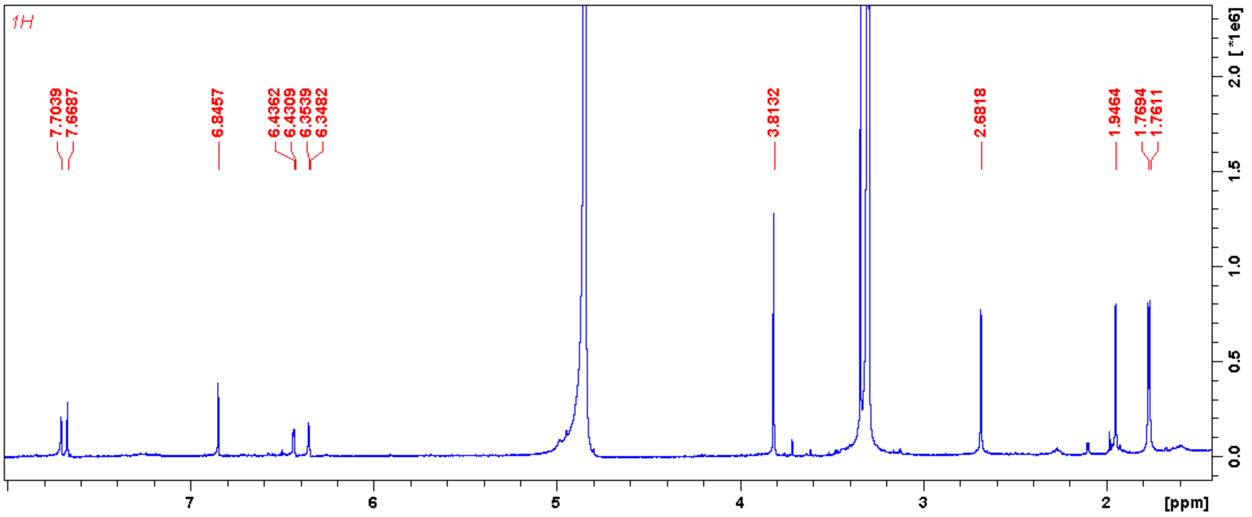

Supplementary Figure 32. ^{1}H NMR spectrum for compound 5. Condition: CD_3OD, 400 MHz.

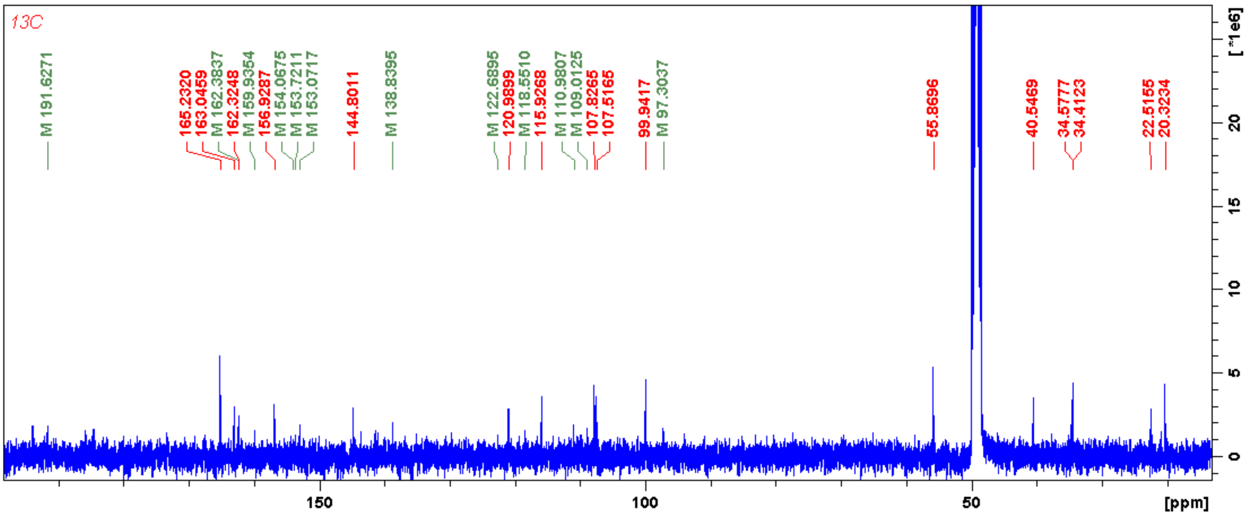

Supplementary Figure 33. ^{13}C NMR spectrum for compound 5. Condition: CD_3OD, 100 MHz.

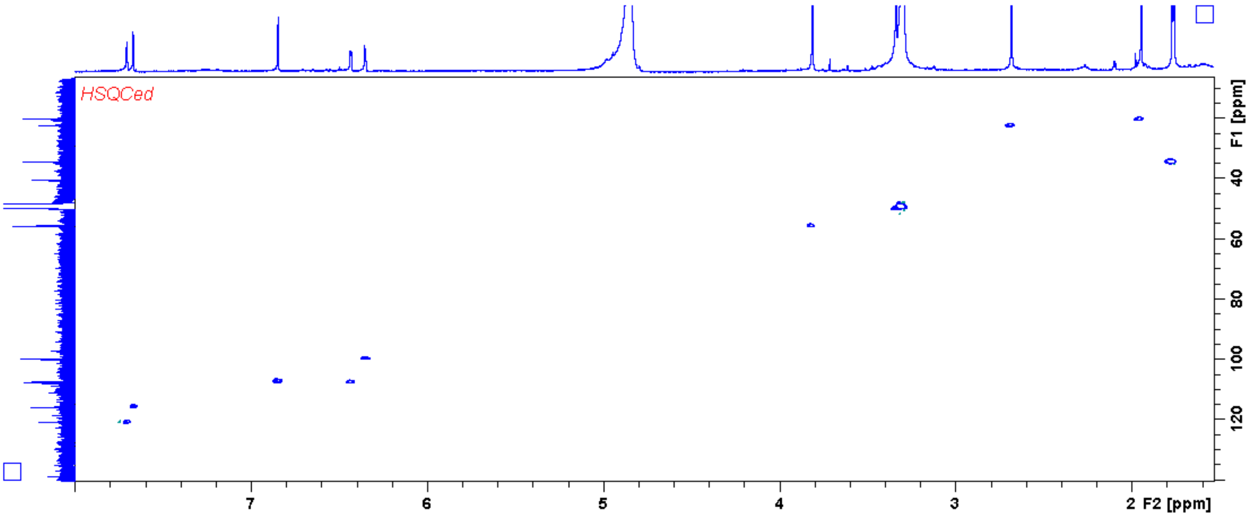

Supplementary Figure 34. HSQC spectrum for compound 5. Condition: CD_3OD.

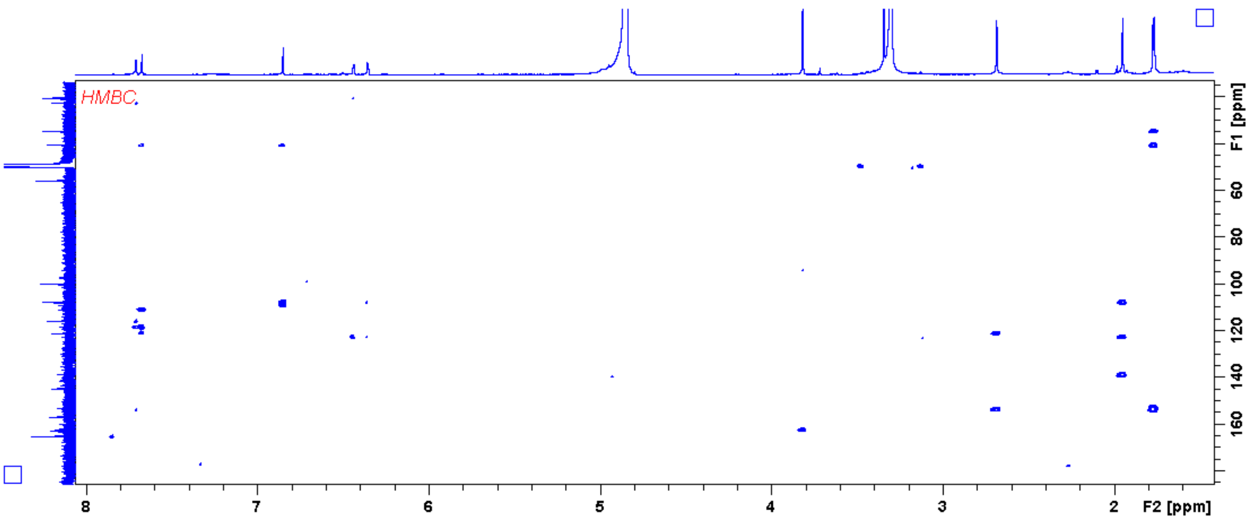

Supplementary Figure 35. HMBC spectrum for compound 5. Condition: CD_3OD.

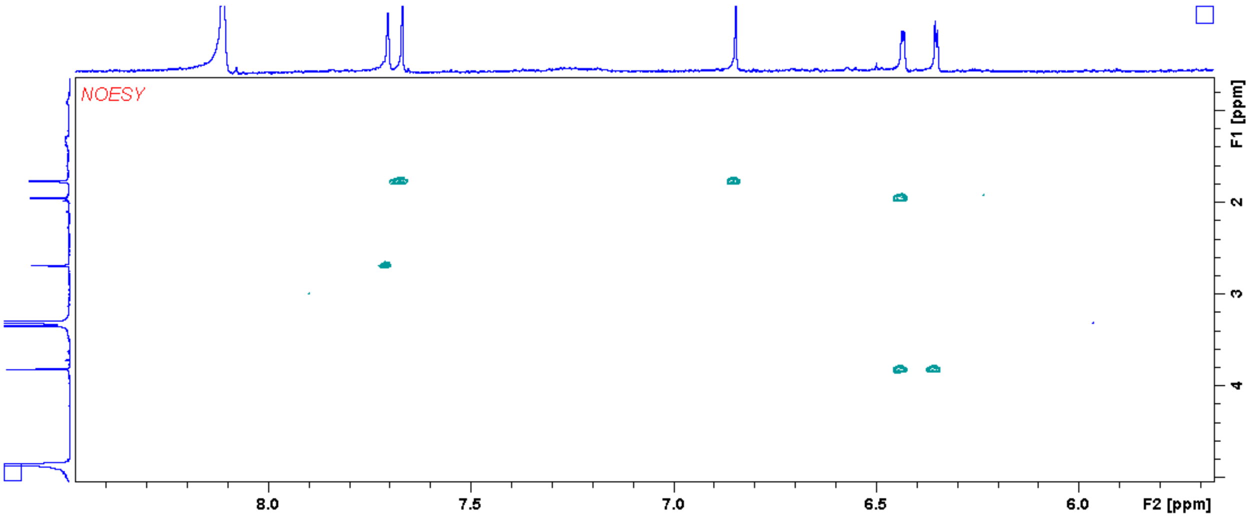

Supplementary Figure 36. NOESY spectrum for compound 5. Condition: CD_3OD.

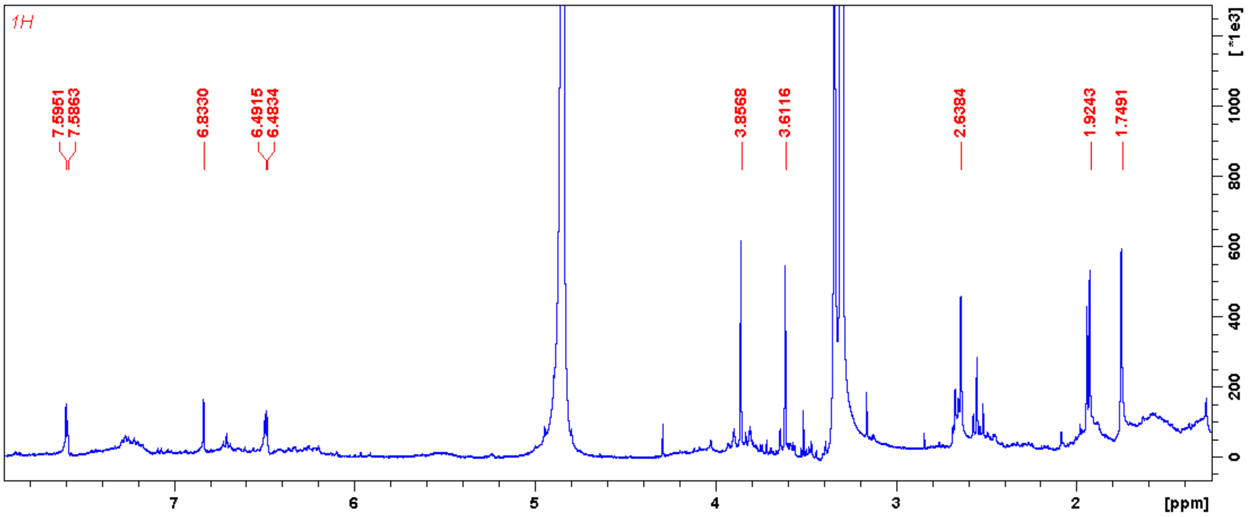

Supplementary Figure 37. ^{1}H NMR spectrum for compound 6. Condition: CD_3OD, 400 MHz.

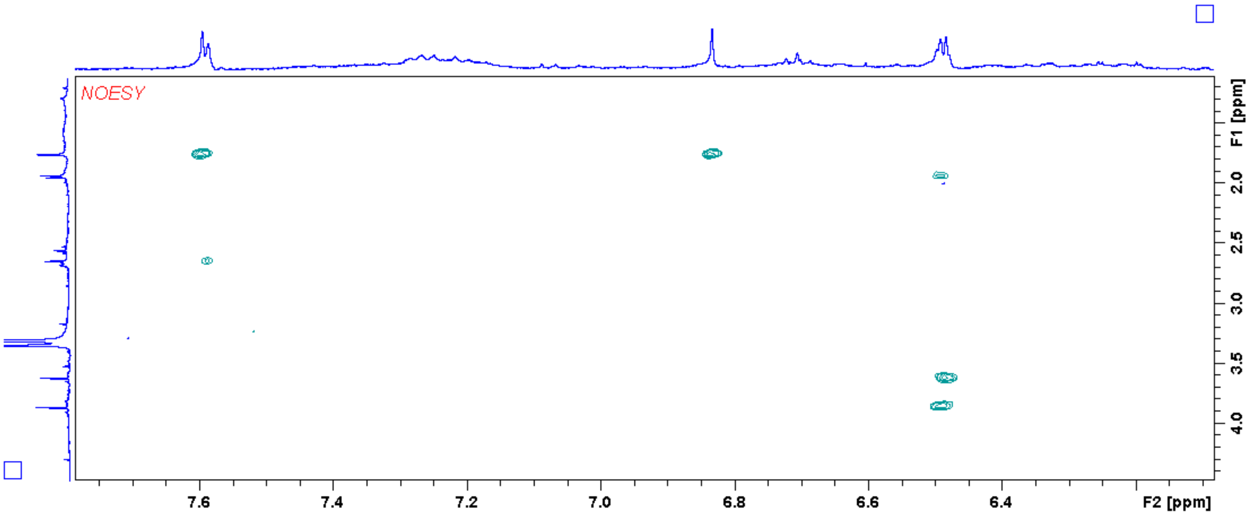

Supplementary Figure 38. NOESY spectrum for compound 6. Condition: CD_3OD.

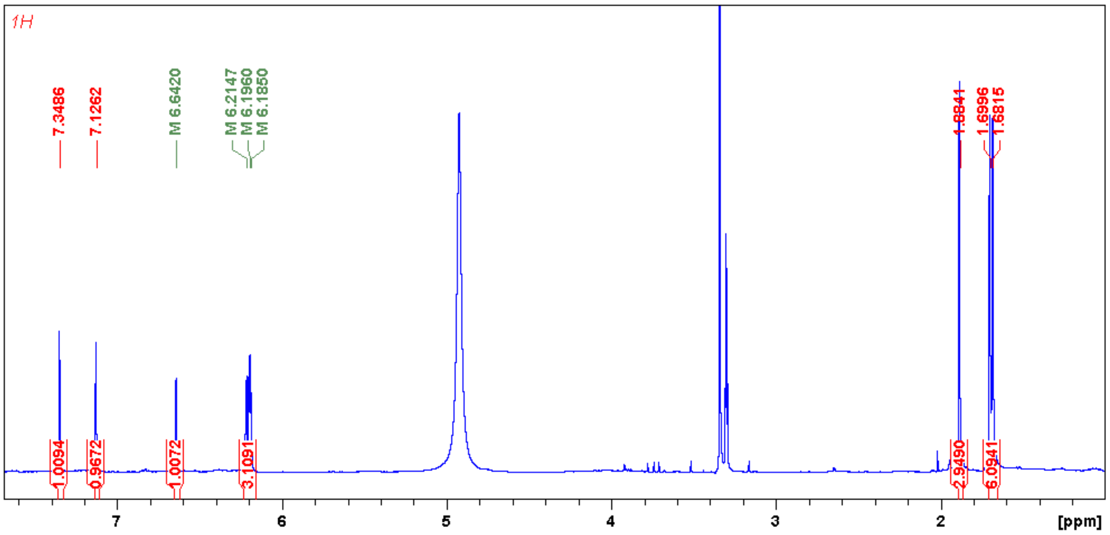

Supplementary Figure 39. ^{1}H NMR spectrum for compound 13. Condition: CD_3OD, 400 MHz.

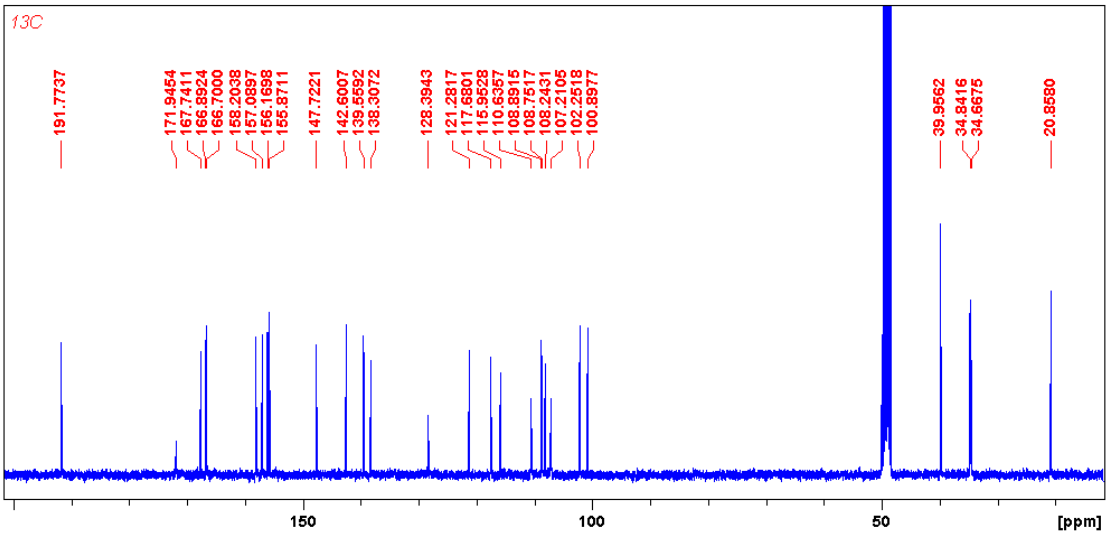

Supplementary Figure 40. ^{13}C NMR spectrum for compound 13. Condition: CD_3OD, 100 MHz.

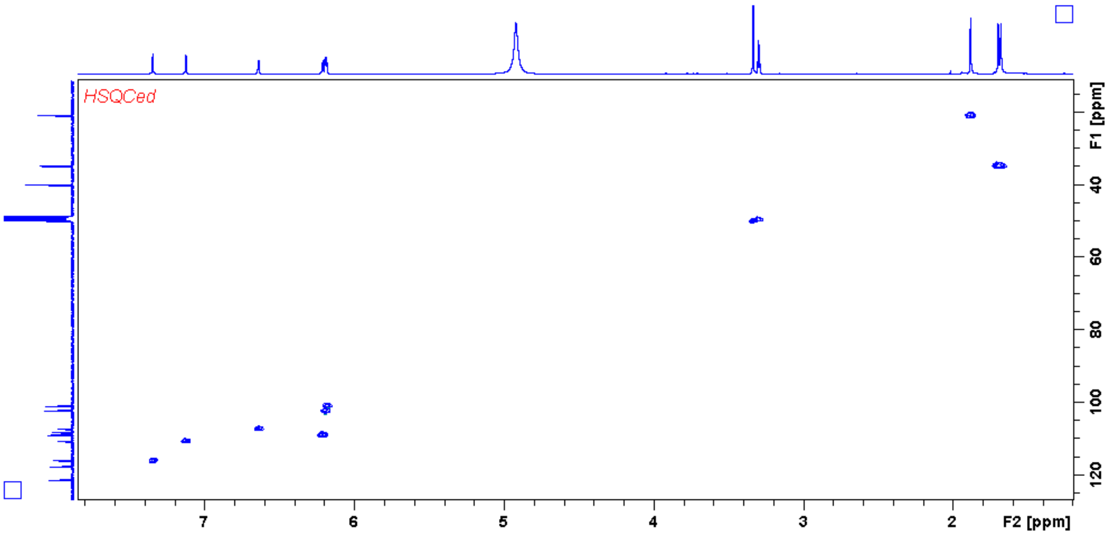

Supplementary Figure 41. HSQC spectrum for compound 13. Condition: CD_3OD.

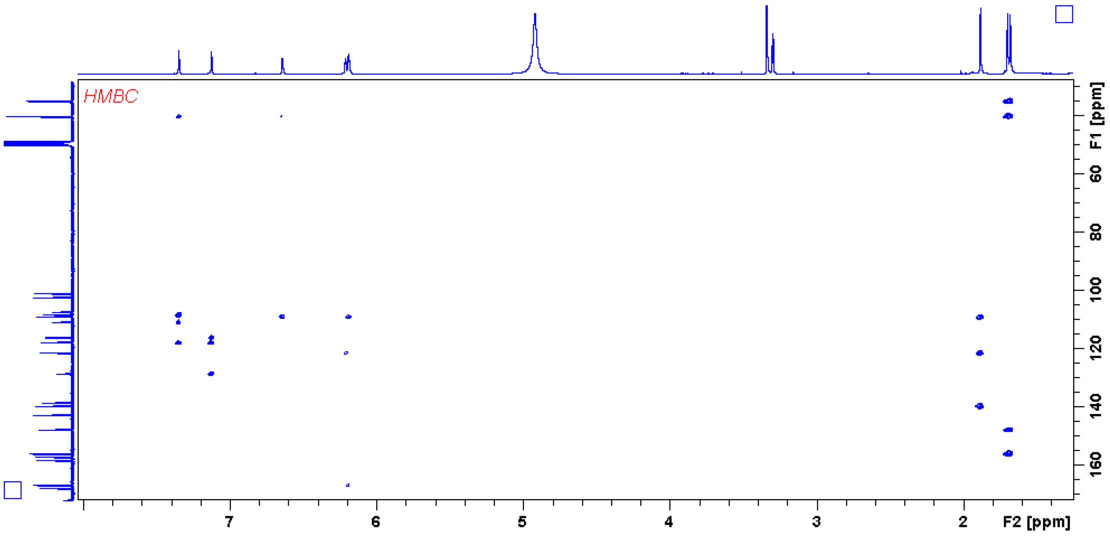

Supplementary Figure 42. HMBC spectrum for compound 13. Condition: CD_3OD.

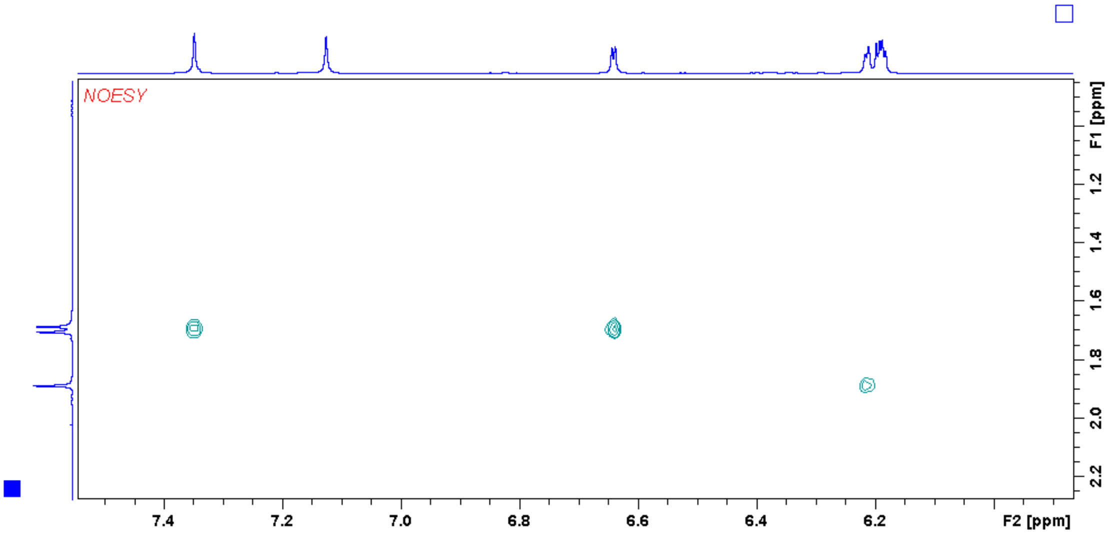

Supplementary Figure 43. NOESY spectrum for compound 13. Condition: CD_3OD.

Supplementary Table 1. Strains made or used in this study.

Strain	Description	Plasmid	Resistance	Source or Reference
E. coli ET12567	*dam*[-] *dcm*[-] *hsdS*[-]	*pUZ8002*	*Cml*[R]/*Tet*[R]	MacNeil *et al.*[1]
E. coli Top10	F– *mcr*A Δ(*mrr-hsd*RMS*mcr*BC) Φ80*lac*ZΔM15 Δ*lac*X74 *rec*A1 *end*A1 *ara*D139 Δ(*ara leu*) 7697 *gal*U *gal*K *rps*L *nup*G			Invitrogen, USA MacNeil *et al.*[1]
E. coli DH10ß	F– *mcr*A Δ(*mrr-hsd*RMS*mcr*BC) Φ80*lac*ZΔM15 Δ*lac*X74 *rec*A1 *end*A1 *ara*D139 Δ(*ara leu*) 7697 *gal*U *gal*K *rps*L *nup*G λ–			Invitrogen, USA
S. formicae	Wild-type strain			MacNeil *et al.*[1]
S. formicae Δ*for*	Formicamycin (*for*) biosynthetic gene cluster (BCG) deleted			MacNeil *et al.*[1]
S. formicae *Δfor:for* ΦC31	*for* BGC deleted and complemented with the *for* BGC in trans at ΦC31	*PESAC-13 215-G*	*Kan*[R]/*Tsr*	MacNeil *et al.*[1]
S. formicae Δ*forV*	Halogenase (*forV*) gene deleted			MacNeil *et al.*[1]
S. *formicae* Δ*forV:forV* *pForV*	Halogenase (*forV*) deleted and complemented at ΦBT1 under the native promoter	pRD004	Hyg[R]	MacNeil *et al.*[1]
S. formicae Δ*forD*	Cyclase (*forD*) gene deleted			This work
S. *formicae*	Cyclase (*forD*) deleted and complemented at	pRD012	Hyg[R]	This work

ΔforD:forD pForD	ΦBT1 under the native promoter			
S. formicae ΔforL	Cyclase (*forL*) gene deleted			This work
S. *formicae ΔforL:forL pForL*	Cyclase (*forL*) deleted and complemented at ΦBT1 under the native promoter	pRD013	Hyg^R	This work
S. formicae ΔforS	Cyclase (*forR*) gene deleted			This work
S. *formicae ΔforS:forS pErmE**	Cyclase (*forR*) deleted and complemented at ΦBT1 under the ErmE* promoter	pRD015	Hyg^R	This work
S. formicae ΔforR	Cyclase (*forS*) gene deleted			This work
S. *formicae ΔforR:forR-A pErmE**	Cyclase (*forS*) deleted and complemented with *forR* and *forA* (directly downstream) at ΦBT1 under the ErmE* promoter	pRD017	Hyg^R	This work
S. formicae ΔforU	Cyclase (*forU*) gene deleted			This work
S. *formicae ΔforU:forU-V pErmE**	Cyclase (*forU*) deleted and complemented with *forU* and *forV* (directly downstream) at ΦBT1 under the ErmE* promoter	pRD019	Hyg^R	This work

Supplementary Table 2. Plasmids and ePACs used in this study.

Plasmids and ePACs	Description	Source or Reference
pCRISPomyces-2	*Streptomyces* plasmid for expression of codon-optimized Cas9 and a single guide RNA	Cobb *et al*.[2]
pUZ8002	Non-transmissible RK2 derivative with a mutation in *oriT*	Kieser *et al*.[3]
pESAC13-215-G	ePAC clone harbouring the *For* BGC integrative ΦC31	MacNeil *et al*.[1]
pMS82	ΦBT1 *attP-int* derived integration vector for the conjugal transfer of DNA from *E. coli* to *Streptomyces* (Hyg^R)	Gregory *et al*.[4]
pIJ10257	Plasmid for the conjugal transfer of DNA (under control of the ermE* constitutive promoter) from *E. coli* to *Streptomyces* spp. Integrates specifically at the ΦBT1 attachment site (HygR)	Hong *et al*.[5]
pRD004	PMS82 pForV ForV complementation plasmid	This work
pRD012	PMS82 pForD ForD complementation plasmid	This work
pRD013	PMS82 pForL ForL complementation plasmid	This work
pRD015	pIJ10257 ForR-ForA complementation plasmid	This work
pRD017	pIJ10257 ForS complementation plasmid	This work
pRD019	pIJ10257 ForU-ForV complementation plasmid	This work

Supplementary Table 3. Growth media used in this study

Media	Recipe (per litre)	Water	pH
LB	10 g tryptone<br>5 g yeast extract<br>10 g NaCl (omitted when selecting with Hygromycin)<br>+/- 20 g agar	Deionised	7.5
MS	20 g soy flour<br>20 g mannitol<br>20 g agar	Tap	As made
MYM	4 g maltose<br>4 g yeast extract<br>10 g malt extract<br>18g agar	50:50<br>Tap:Deisonised	7.3

Supplementary Table 4. Antibiotics used in this study.

Antibiotic	Final concentration used for selection (µg ml^{-1})
Apramycin	50
Hygromycin	50
Kanamycin	50
Chloramphenicol	30
Nalidixic Acid	25

Supplementary Note 1. Characterization of compounds reported in this study

6-chlorogenistein. Yield: 2 mg; UV/Vis: λ_{max} = 259 nm; Molecular formula: $C_{15}H_9O_5Cl$; HRMS (ESI) *m/z*: calculated $[M + H]^+$ = 305.0211, observed $[M + H]^+$ = 305.0212, Δ = 0.33 ppm; ^{1}H NMR (400 MHz, CD_3OD): δ 8.2 (s, 1H, 2-*H*), 7.4 (s, 1H, 3'-*H*), 7.4 (s, 1H, 5'-*H*), 6.9 (s, 1H, 6'-*H*), 6.8 (s, 1H, 2'-*H*), 6.4 (s, 1H, 8-*H*); ^{13}C NMR (100 MHz, CD_3OD): δ 182.3 (*C*-4), 162.2 (*C*-5), 162.0 (*C*-7), 159.2 (*C*-4'), 155.0 (*C*-8a), 155.0 (*C*-2), 131.6 (*C*-3'), 131.6 (*C*-5'), 125.2 (*C*-1'), 123.1 (*C*-3), 116.5 (*C*-2'), 116.5 (*C*-6'), 107.0 (*C*-4A), 100.5 (*C*-8), 99.5 (*C*-6). An isomer of 6-chlorogenistein, identified based on based on UV and MS characteristics, and likely to be a chlorination regioisomer, was also identified in the LCMS trace but was not isolated due to low levels of production. The NMR data for 6-chlorogenistein is in accordance with that reported previously[6].

Formicapyridine A (**1**). Yield: 1 mg; UV/Vis: λ_{max} = 228, 249, 272 and 391 nm; specific rotation: $[\alpha]^{20}_D$ = +9.9 (c = 0.09, methanol); Molecular formula: $C_{27}H_{23}NO_6$; HRMS (ESI) *m/z*: calculated $[M + H]^+$ = 458.1598, observed $[M + H]^+$ = 458.1600, Δ = 0.44 ppm; ^{1}H NMR (400 MHz, CD_3OD): δ 7.7 (s, 1H, 20-*H*), 7.7 (s, 1H, 22-*H*), 6.7 (d, J = 2.21 Hz, 1H, 16-*H*), 6.3 (d, J = 2.21 Hz, 1H, 2-*H*), 6.3 (d, J = 1.64 Hz, 1H, 4-*H*), 6.3 (d, J = 1.64 Hz, 1H, 14-*H*), 2.7 (s, 3H, 25-*H*), 1.9 (s, 3H, 24-*H*), 1.8 (s, 3H, 26-*H*), 1.8 (s, 3H, 27-*H*); ^{13}C NMR (100 MHz, CD_3OD): δ 191.6 (*C*-11), 167.8 (*C*-15), 167.7 (*C*-13), 167.5 (*C*-9), 159.9 (*C*-7), 157.0 (*C*-5), 155.7 (*C*-17), 155.1 (*C*-19), 152.6 (*C*-23), 144.7 (*C*-21), 138.8 (*C*-1), 121.3 (*C*-22), 120.9 (*C*-6), 118.6 (*C*-8), 115.7 (*C*-20), 111.3 (*C*-10), 109.4 (*C*-2), 108.5 (*C*-12), 107.9 (*C*-16), 102.5 (*C*-14), 101.2 (*C*-4), 40.7 (*C*-18), 34.7 (*C*-27), 34.5 (*C*-26), 22.1 (*C*-25), 20.2 (*C*-24).

Formicapyridine B (**2**). Yield: 2 mg; UV/Vis: λ_{max} = 229, 249, 272 and 392 nm; specific rotation: $[\alpha]^{20}_D$ = +7.7 (c = 0.18, methanol); Molecular formula: $C_{28}H_{25}NO_6$; HRMS (ESI) *m/z*: calculated $[M + H]^+$ = 472.1755, observed $[M + H]^+$ = 472.1753, Δ = -0.42 ppm; ^{1}H NMR (400 MHz, CD_3OD): δ 7.6 (s, 1H, 22-*H*), 7.6 (s, 1H, 20-*H*), 6.7 (d, J = 2.25 Hz, 1H, 16-*H*), 6.4 (d, J = 2.27 Hz, 1H, 2-*H*), 6.3 (d, J = 2.27 Hz, 1H, 4-*H*), 6.2 (d, J = 2.25 Hz, 1H, 14-*H*), 3.8 (s, 3H, 28-*H*), 2.7 (s, 3H, 25-*H*), 1.9 (s, 3H, 24-*H*), 1.8 (s, 3H, 26-*H*), 1.7 (s, 3H, 17-*H*); ^{13}C NMR (100 MHz, CD_3OD): δ 191.8 (*C*-11), 167.5 (*C*-13), 167.4 (*C*-15), 167.3 (*C*-9), 161.7 (*C*-3), 160.1 (*C*-7), 156.7 (*C*-5), 155.8 (*C*-17), 155.5 (*C*-23), 152.7 (*C*-19), 144.4 (*C*-21), 138.6 (*C*-1), 124.9 (*C*-6), 119.9 (*C*-22), 118.6 (*C*-8), 115.6 (*C*-20), 110.6 (*C*-10), 108.6 (*C*-12), 107.7 (*C*-2), 107.6 (*C*-16), 102.5 (*C*-14), 99.9 (*C*-4), 55.8 (*C*-28), 40.5 (*C*-18), 34.8 (*C*-27), 34.6 (*C*-26), 23.6 (*C*-25), 20.4 (*C*-24).

Formicapyridine C (**3**). Yield: 2 mg; UV/Vis: λ_{max} = 228, 249, 272 and 391 nm; specific rotation: $[\alpha]^{20}_{D}$ = +8.8 (c = 0.18, methanol); Molecular formula: $C_{29}H_{27}NO_6$; HRMS (ESI) *m/z*: calculated $[M + H]^+$ = 486.1911, observed $[M + H]^+$ = 486.1905, Δ = -1.23 ppm; ^1H NMR (400 MHz, CD_3OD): δ 7.6 (s, 1H, 22-*H*), 7.6 (s, 1H, 20-*H*), 6.7 (d, J = 2.30 Hz, 1H, 16-*H*), 6.5 (d, J = 2.33 Hz, 1H, 2-*H*), 6.5 (d, J = 2.33 Hz, 1H, 4-*H*), 6.2 (d, J = 2.30 Hz, 1H, 14-*H*), 3.9 (s, 3H, 28-*H*), 3.6 (s, 3H, 29-*H*), 2.6 (s, 3H, 25-*H*), 1.9 (s, 3H, 24-*H*), 1.8 (s, 3H, 27-*H*), 1.7 (s, 3H, 26-*H*); ^{13}C NMR (100 MHz, CD_3OD): δ 191.9 (*C*-11), 167.7 (*C*-15), 167.4 (*C*-13), 167.2 (*C*-9), 162.4 (*C*-3), 161.0 (*C*-7), 159.7 (*C*-5), 155.8 (*C*-17), 154.5 (*C*-23), 153.6 (*C*-19), 144.4 (*C*-21), 138.5, (*C*-1) 125.1 (*C*-6), 120.4 (*C*-22), 118.4 (*C*-8), 115.7 (*C*-20), 110.8 (*C*-10), 108.5 (*C*-12),107.8 (*C*-16), 107.7 (*C*-2), 102.5 (*C*-14), 97.0 (*C*-4), 56.3 (*C*-29), 56.0 (*C*-28), 40.6 (*C*-18), 34.6 (*C*-26), 34.6 (*C*-27), 23.1 (*C*-25), 20.2 (*C*-24).

Formicapyridine D (**4**). Yield: 0.7 mg; UV/Vis: λ_{max} = 228, 252, and 391 nm; specific rotation: $[\alpha]^{20}_{D}$ = +11.0 (c = 0.06, methanol); Molecular formula: $C_{27}H_{22}NO_6Cl$; HRMS (ESI) *m/z*: calculated $[M + H]^+$ = 492.1218, observed $[M + H]^+$ = 492.1208, Δ = 2.03 ppm; ^1H NMR (400 MHz, CD_3OD): δ 7.6 (s, 1H, 22-*H*), 7.6 (s, 1H, 20-*H*), 6.8 (s, 1H, 16-*H*), 6.3 (d, J = 2.32 Hz, 1H, 2-*H*), 6.3 (d, J = 2.32 Hz, 1H, 4-*H*), 2.6 (s, 3H, 25-*H*), 1.9 (s, 3H, 24-*H*), 1.7 (s, 3H, 26-*H*), 1.7 (s, 3H, 27-*H*).

Formicapyridine E (**5**). Yield: 1 mg; UV/Vis: λ_{max} = 218, 252, and 391 nm; specific rotation: $[\alpha]^{20}_{D}$ = +12.1 (c = 0.09, methanol); Molecular formula: $C_{28}H_{24}NO_6Cl$; HRMS (ESI) *m/z*: calculated $[M + H]^+$ = 506.1365, observed $[M + H]^+$ = 506.1362, Δ = -0.59 ppm; ^1H NMR (400 MHz, CD_3OD): δ 7.7 (s, 1H, 22-*H*), 7.7 (s, 1H, 20-*H*), 6.8 (d, 1H, 16-*H*), 6.4 (d, J = 2.22 Hz, 1H, 2-*H*), 6.3 (d, J = 2.22 Hz, 1H, 4-*H*), 3.8 (s, 3H, 28-*H*), 2.7 (s, 3H, 25-*H*), 1.9 (s, 3H, 24-*H*), 1.7 (s, 3H, 26-*H*), 1.7 (s, 3H, 27-*H*); ^{13}C NMR (100 MHz, CD_3OD): δ 191.6 (*C*-11), 165.2 (*C*-13), 163.0 (*C*-15), 162.4 (*C*-3), 162.3 (*C*-9), 159.9 (*C*-7), 156.9 (*C*-5), 154.1 (*C*-17), 153.7 (*C*-23), 153.1 (*C*-19), 144.8 (*C*-21), 138.8 (*C*-1), 122.7 (*C*-6), 121.0 (*C*-22), 118.6 (*C*-8), 115.9 (*C*-20), 111.0 (*C*-10), 109.0 (*C*-12), 107.8 (*C*-2), 107.5 (*C*-16), 99.9 (*C*-4), 97.3 (*C*-14), 55.9 (*C*-28), 40.5 (*C*-18), 34.6 (*C*-27), 34.4 (*C*-26), 22.5 (*C*-25), 20.3 (*C*-24).

Formicapyridine F (**6**). Yield: 0.6 mg; UV/Vis: λ_{max} = 231, 252, and 391 nm; specific rotation: $[\alpha]^{20}_{D}$ = +12.8 (c = 0.06, methanol); Molecular formula: $C_{29}H_{26}NO_6Cl$; HRMS (ESI) *m/z*: calculated $[M + H]^+$ = 520.1521, observed $[M + H]^+$ = 520.1525, Δ = 0.77 ppm; ^1H NMR (400 MHz, CD_3OD): δ 7.6 (s, 1H, 22-*H*), 7.6 (s, 1H, 20-*H*), 6.8 (d, 1H, 16-*H*), 6.5 (d, J = 2.33 Hz, 1H, 2-*H*), 6.5 (d, J = 2.33 Hz, 1H, 4-*H*), 3.9 (s, 3H, 28-*H*), 3.6 (s, 3H, 29-*H*), 2.6 (s, 3H, 25-*H*), 1.9 (s, 3H, 24-*H*), 1.7 (s, 3H, 26-*H*), 1.7 (s, 3H, 27-*H*).

Fasamycin F (**13**). Yield: 3.4 mg; UV/Vis: λ_{max} = 247, 287, 357 and 414 nm; specific rotation: $[\alpha]^{20}_{D}$ = 4.3 (0.54, methanol); Molecular formula: $C_{28}H_{29}O_9$; HRMS (ESI) *m/z*: calculated $[M + H]^+$ = 503.1337, observed $[M + H]^+$ = 503.1326, Δ = -0.29 ppm; ^{1}H NMR (400 MHz, CD_3OD): δ 7.3 (s, 1H, 20-*H*), 7.1 (s, 1H, 22-*H*), 6.6 (d, J = 2.22 Hz, 1H, 16-*H*), 6.2 (d, J = 2.22 Hz, 1H, 14-*H*), 6.2 (d, J = 2.30 Hz, 1H, 2-*H*), 6.2 (d, J = 2.30 Hz, 1H, 4-*H*), 1.9 (s, 3H, 28-*H*), 1.7 (s, 3H, 25-*H*), 1.7 (s, 3H, 26-*H*); ^{13}C NMR (100 MHz, CD_3OD): δ 191.8 (*C*-11), 172.0 (*C*-27), 167.7 (*C*-9), 166.9 (*C*-13), 166.7 (*C*-15), 158.2 (*C*-3), 157.1 (*C*-23), 156.2 (*C*-5), 155.9 (*C*-17), 147.7 (*C*-19), 142.6 (*C*-21), 139.6 (*C*-1), 138.3 (*C*-7), 128.4 (*C*-24), 121.3 (*C*-6), 117.7 (*C*-8), 116.0 (*C*-20), 110.6 (*C*-22), 108.9 (*C*-2), 108.8 (*C*-12), 108.2 (*C*-10), 107.2 (*C*-16), 102.3 (*C*-14), 100.9 (*C*-4), 40.0 (*C*-18), 34.8 (*C*-26), 34.7 (*C*-25), 20.9 (*C*-28).

Supplementary References

[1] MacNeil, D. J., Gewain, K. M., Ruby, C. L., Dezeny, G., Gibbons, P. H., MacNeil, T. Analysis of *Streptomyces avermitilis* genes required for avermectin biosynthesis utilizing a novel integration vector. *Gene*. **111**, 61-68 (1992).

[2] Cobb, R. E., Wang, Y. & Zhao, H. High-efficiency multiplex genome editing of *Streptomyces* species using and engineered CRISPR/Cas system. *ACS Synth. Biol*. **4**, 723-728 (2015).

[3] Kieser, T., Bibb, M. J., Buttner, M. J., Chater, K. F., Hopwood, D. A. *Practical Streptomyces Genetics* (The John Innes Foundation Press, Norwich, 2000).

[4] Gregory, M. A., Till, R., Smith, M. C. M. Integration site for *Streptomyces* phage φBT1 and development of site-specific integrating vectors. *J Bacteriol.* **185**, 5320-5323 (2003).

[5] Hong, H. J., Hutchings, M. I., Hill, L. M., Buttner, M. J. The role of the novel fem protein VanK in vancomycin resistance in *Streptomyces coelicolor*. *J. Biol. Chem.* **280**, 13055-13061 (2005).

[6] Konig, W. A., Krauss, C., Zahner, H. Metabolites from microorganisms, 6-chlorogenistein and 6,3'-dichlorogenistein. *Helv. Chim. Acta*. **60**, 2071–2078 (1977).
