# Supplementary material for: A role for antibiotic biosynthesis monooxygenase domain proteins in fidelity control during aromatic polyketide biosynthesis
Source: Nat Commun. 2019 Aug 9;10:3611. doi: 10.1038/s41467-019-11538-6 (PMC6689052; doi:10.1038/s41467-019-11538-6)
Supplement: Supplementary file 3 — Reporting Summary [file 41467_2019_11538_MOESM3_ESM.pdf]

nature research

Corresponding author(s): Barrie Wilkinson and Mathew I Hutchings
NCOMMS-19-07612

Last updated by author(s): Jul 5, 2019

Reporting Summary

Nature Research wishes to improve the reproducibility of the work that we publish. This form provides structure for consistency and transparency in reporting. For further information on Nature Research policies, see Authors & Referees and the Editorial Policy Checklist.

Statistics

For all statistical analyses, confirm that the following items are present in the figure legend, table legend, main text, or Methods section.

n/a	Confirmed	
☐	☒	The exact sample size (n) for each experimental group/condition, given as a discrete number and unit of measurement
☐	☒	A statement on whether measurements were taken from distinct samples or whether the same sample was measured repeatedly
☒	☐	The statistical test(s) used AND whether they are one- or two-sided<br>*Only common tests should be described solely by name; describe more complex techniques in the Methods section.*
☒	☐	A description of all covariates tested
☐	☒	A description of any assumptions or corrections, such as tests of normality and adjustment for multiple comparisons
☐	☒	A full description of the statistical parameters including central tendency (e.g. means) or other basic estimates (e.g. regression coefficient) AND variation (e.g. standard deviation) or associated estimates of uncertainty (e.g. confidence intervals)
☒	☐	For null hypothesis testing, the test statistic (e.g. F, t, r) with confidence intervals, effect sizes, degrees of freedom and P value noted<br>*Give P values as exact values whenever suitable.*
☒	☐	For Bayesian analysis, information on the choice of priors and Markov chain Monte Carlo settings
☒	☐	For hierarchical and complex designs, identification of the appropriate level for tests and full reporting of outcomes
☒	☐	Estimates of effect sizes (e.g. Cohen's d, Pearson's r), indicating how they were calculated

Our web collection on statistics for biologists contains articles on many of the points above.

Software and code

Policy information about availability of computer code

Data collection	LCMS(UV) data was collected using Shimadzu LCMSsolution v.3.80.
Data analysis	LCMS(UV) raw data was analyzed using Shimadzu LCMSsolution v.3.80.<br>LCMS datasets were analyzed/processed using Shimadzu Profiling Solutions v.1.1.(this is noted in the manuscript).<br>No further software other than Microsoft exel was used for data collection, manipulation and analysis.

For manuscripts utilizing custom algorithms or software that are central to the research but not yet described in published literature, software must be made available to editors/reviewers. We strongly encourage code deposition in a community repository (e.g. GitHub). See the Nature Research guidelines for submitting code & software for further information.

Data

Policy information about availability of data

All manuscripts must include a data availability statement. This statement should provide the following information, where applicable:

- Accession codes, unique identifiers, or web links for publicly available datasets
- A list of figures that have associated raw data
- A description of any restrictions on data availability

The authors declare that the data supporting the findings reported in this study are available within the article and the Supplementary Information or are available from the authors on reasonable request.

Field-specific reporting

Please select the one below that is the best fit for your research. If you are not sure, read the appropriate sections before making your selection.

☒ Life sciences ☐ Behavioural & social sciences ☐ Ecological, evolutionary & environmental sciences

For a reference copy of the document with all sections, see nature.com/documents/nr-reporting-summary-flat.pdf

Life sciences study design

All studies must disclose on these points even when the disclosure is negative.

Sample size	Our fermentation experiments were each run in triplicate using three independent fermentations.
Data exclusions	No data was excluded.
Replication	There were no examples of replicate experiments failing and we describe all examples of where an experiment was modified and repeated e.g. when a polar effect in a mutant was observed we repeated the experiment and ectopically expressed a copy of the affected gene. This data is then reported.
Randomization	Each mutant was analyzed in triplicate by using three independent biological replicates. These were the first three colonies picked from an experiment and they were picked randomly from selection plates.
Blinding	All relevant lab work was carried out by the same two scientists and they could not blind themselves.

Reporting for specific materials, systems and methods

We require information from authors about some types of materials, experimental systems and methods used in many studies. Here, indicate whether each material, system or method listed is relevant to your study. If you are not sure if a list item applies to your research, read the appropriate section before selecting a response.

Materials & experimental systems

n/a	Involved in the study
☒	☐ Antibodies
☒	☐ Eukaryotic cell lines
☒	☐ Palaeontology
☒	☐ Animals and other organisms
☒	☐ Human research participants
☒	☐ Clinical data

Methods

n/a	Involved in the study
☒	☐ ChIP-seq
☒	☐ Flow cytometry
☒	☐ MRI-based neuroimaging
