# Supplementary material for: A role for antibiotic biosynthesis monooxygenase domain proteins in fidelity control during aromatic polyketide biosynthesis
Source: Nat Commun. 2019 Aug 9;10:3611. doi: 10.1038/s41467-019-11538-6 (PMC6689052; doi:10.1038/s41467-019-11538-6)
Supplement: Supplementary file 4 — Description of Additional Supplementary Files [file 41467_2019_11538_MOESM4_ESM.docx]

**Description of Additional Supplementary Files**

File Name: Supplementary Data 1
Description: Source data extracted from LCMS experiments using the Shimadzu Profiling Solution software

File Name: Supplementary Data 2
Description: Targeted metabolomic analysis identifies the formicapyridines A-F

File Name: Supplementary Data 3
Description: Standards calibration data

File Name: Supplementary Data 4
Description: Comparative and quantitative analysis of target metabolites

File Name: Supplementary Data 5
Description: PCR primers used in this study
