# Supplementary material for: A role for antibiotic biosynthesis monooxygenase domain proteins in fidelity control during aromatic polyketide biosynthesis
Source: Nat Commun. 2019 Aug 9;10:3611. doi: 10.1038/s41467-019-11538-6 (PMC6689052; doi:10.1038/s41467-019-11538-6)
Supplement: Supplementary file 2 — Peer Review File [file 41467_2019_11538_MOESM2_ESM.pdf]

Reviewers' comments:

Reviewer #1 (Remarks to the Author):

This interesting manuscript describes carefully done work to elucidate the structures of nine formicapyridines and fasamycin F produced by Streptomyces formicae. The former compounds (not antimicrobially active) are shown to be shunt polyketide metabolites whose production by the PKS systems normally making fasamycins can be enhanced by deletion of the forS gene. The formation of pyridine ring containing structures by polyketide synthase systems is relatively rare. The work is demanding, carefully done, fully described and merits publication after some minor considerations......
On page 8 and in Figure 4 the authors propose an aminotransferase catalyzed transamination, which would the be followed by cyclization and oxidation. However, simple addition of ammonia to the ketone to form an imine, tautomerization to an enamine and subsequent cyclization could give the required product without an oxidation step. This could be mentioned in the manuscript as an alternative pathway......
On page 15 line 357 the authors have written BCG when they mean BGC.......
Throughout: The manuscript is not as concise as it could be. The authors use many phrases that add nothing to the meaning and expand the text. Examples of this would be: "We speculate, tentatively, that..." "It should be noted that..." If the authors were to go through the manuscript and remove every non-essential phrase or statement, the resulting shorter manuscript would have a better impact.

Reviewer #2 (Remarks to the Author):

This is an interesting, well carried out, and well discussed contribution by Wilkinson et al., and should be accepted.
The work clearly goes beyond the discovery of formerly undiscovered or overlooked side products, since the authors could clearly and convincingly show that the lack of a fourth cyclase activity combined with a transamination affecting one of the "sticking out" keto groups lead to the unusual formicapyridine scaffold, and they also could re-program the pathway towards accumulation of these novel products. However, these products are artifacts, due to a substrate flexible transaminase outside the BCG, and not surprisingly biologically inactive.

Some remarks:

Besides the unassigned carbonyl and phenol carbon mentioned in lines 147/148, C-7 also has no connectivity assignment from HMBC.

Why bolding the 'eastern half' of ring A of the fasamycins and formicamycins, if the stereochemistry resulting form the atrop-isomerism is unknown (line 161, vs structures Fig 1)

The 1,2-13C2-acetate feeding experiment does not really prove anything, since C-25 would be enriched no matter whether derived from C-1 or C-2 of acetate. The text is a little misleading here. However, the authors proved that the decarboxylation occured at C-25 (singlet in contrast to C-24, which shows coupling to C-1), which proves the incorporation pattern of acetate units as shown in Fig. 5. BTW: C-25 hardly looks enriched (comparison of the red vs the green 13C NMR spectrum, Fig 5)

Please label all peaks in Fig. 6, i.e. compounds other than formicapyridines in traces b, c, d.

There are some inconsistencies and typos in the text and references. All errors I could find are marked yellow and with sticky notes in the manuscript.

Jurgen Rohr

Reviewer #3 (Remarks to the Author):

In this manuscript, the authors identified the formicapyridines, pyridine containing polyketides alkaloids which represent additional products of the formicamycin biosynthetic gene cluster, by the aid of targeted metabolomics. The amount of these compounds is at least 100-fold lower than the major compounds, so it is challenging for isolation and structural identification. Subsequent mutation experiments indicated that the productivity of formicapyridines were increased in the antibiotic biosynthetic monooxygenase decode forS–deletion mutant, while the major products titer was decreased. According to these results, they speculate that ForS had the non-catalytic function but maintaining biosynthetic pathway fidelity in the biosynthetic pathway. While, more experiments need to be done before publication, including some suggestions/questions as following:
1. Title is too vague and broad, which likes a review. Importantly, there are no many works on evolution and engineering, both of which are not sufficiently expressed in the text.
2. Cyclases were well discussed, but there is no supportive data and meaningful results in discussion. Although it is interesting that the deletion of forS promotes the production of formicapyridines, there is no further research to be carried out to illuminate its function. The possible biosynthesis of formicapyridine backbone was only proposed, but the transamination was not systematically analyzed.
3. The authors mentioned that the stereochemistry of compounds 1-6 were unassigned because of the small amounts and very weak electronic dichroism. Indeed, the productivity of these formicapyridines is very low in WT strain, but the titer was increased approximate 25-fold in the ΔforS mutant, which provide a chance to determine the stereochemistry. So, I think enough compounds should be obtained to assign the stereochemistry.
4. There are some impurity peaks and peaks with weak signal in NMR data, such as supplementary Figure 18 and supplementary Figure 30. In supplementary Figure 10, there should be more data points (at least 5) to be collected to draw calibration curves. Also, experiments should be repeated three times to obtain error bar.
5. Page 15, line 365, please cite references for PKS cyclases as fusion proteins with an ABM domain; in addition, in regard to the function of ABM, is it possible to distinguish chaperone-like function and enzymatic function in nucleotide sequence or amino sequence?
6. In Figure 6a and Supplementary Figure 6f, there is a unlabeled peak at ca. 9.8 min, is it related with formicapyridines or formicamycin?
7. Is there any gene encoded potential transaminase in for gene cluster or in the genome? If that, overexpression of it may enhance the productivity of formicapyridines.
8. Minor mistakes: (a) TEXT, in the legend of Figure 3, change "NOSEY (red double head 166 arrows)" to "NOSEY (red double headed 166 arrows)"; (b) in Electronic SI, P40, change "COMPOUND 15 (fasamycin F)" to "COMPOUND 13 (fasamycin F)"; (c) the labels of each chromatogram in Supplementary Figure 9 is not consistent with their legend, please check it again.

**NCOMMS-19-07612 revisions**

Dear Chuanfu,

Please find our detailed response (in green) to the three sets of reviewer comments.

Best regards,

Barrie

**Reviewer 1:**

This interesting manuscript describes carefully done work to elucidate the structures of nine formicapyridines and fasamycin F produced by Streptomyces formicae. The former compounds (not antimicrobially active) are shown to be shunt polyketide metabolites whose production by the PKS systems normally making fasamycins can be enhanced by deletion of the forS gene. The formation of pyridine ring containing structures by polyketide synthase systems is relatively rare. The work is demanding, carefully done, fully described and merits publication after some minor considerations......

We appreciate the positive response of this reviewer.

On page 8 and in Figure 4 the authors propose an aminotransferase catalyzed transamination, which would the be followed by cyclization and oxidation. However, simple addition of ammonia to the ketone to form an imine, tautomerization to an enamine and subsequent cyclization could give the required product without an oxidation step. This could be mentioned in the manuscript as an alternative pathway......

We thank the reviewer for their mechanism suggestion and have amended the text to include for this possibility. We have also included some bioinformatic analysis and discussion regarding the aminotransferase complement of the organism in response to this comment and the comments of Reviewer 3 below.

On page 15 line 357 the authors have written BCG when they mean BGC.......

This typo has been corrected.

Throughout: The manuscript is not as concise as it could be. The authors use many phrases that add nothing to the meaning and expand the text. Examples of this would be: "We speculate, tentatively, that..." "It should be noted that..." If the authors were to go through the manuscript and remove every non-essential phrase or statement, the resulting shorter manuscript would have a better impact.

We have done our best to edit the manuscript in line with these comments and generally streamline text where appropriate. All changes are tracked.

**Reviewer 2:**

This is an interesting, well carried out, and well discussed contribution by Wilkinson et al., and should be accepted.

The work clearly goes beyond the discovery of formerly undiscovered or overlooked side

products, since the authors could clearly and convincingly show that the lack of a fourth cyclase activity combined with a transamination affecting one of the "sticking out" keto groups lead to the unusual formicapyridine scaffold, and they also could re-program the pathway towards accumulation of these novel products. However, these products are artifacts, due to a substrate flexible transaminase outside the BCG, and not surprisingly biologically inactive.

We appreciate the positive comments from Jurgen.

Some remarks:
Besides the unassigned carbonyl and phenol carbon mentioned in lines 147/148, C-7 also has no connectivity assignment from HMBC.

We have edited the sentence to clarify this omission.

Why bolding the 'eastern half' of ring A of the fasamycins and formicamycins, if the stereochemistry resulting form the atrop-isomerism is unknown (line 161, vs structures Fig 1).

We have removed this bolding from the formicamycin structures as for this set of molecules the atropoisomer was previously implied from the fasamycin structure, where we were able to verify which isomer is in place (see Qin et al, Chem Sci 2017).

The 1,2-13C2-acetate feeding experiment does not really prove anything, since C-25 would be enriched no matter whether derived from C-1 or C-2 of acetate. The text is a little misleading here. However, the authors proved that the decarboxylation occured at C-25 (singlet in contrast to C-24, which shows coupling to C-1), which proves the incorporation pattern of acetate units as shown in Fig. 5. BTW: C-25 hardly looks enriched (comparison of the red vs the green 13C NMR spectrum, Fig 5).

The text has been modified slightly so that we (hopefully) are not overreaching our data. However, there is definite enrichment for this carbon which, with the lack of coupling for C25, shows it derives from an acetate unit.

Please label all peaks in Fig. 6, i.e. compounds other than formicapyridines in traces b, c, d.

The peaks have been labelled with an asterisk and the legend modified to clarify that these compounds are unrelated to the for pathway based on UV and *m/z* analysis.

There are some inconsistencies and typos in the text and references. All errors I could find are marked yellow and with sticky notes in the manuscript.

Jurgen Rohr

The various typos have been corrected.

**Reviewer 3:**

In this manuscript, the authors identified the formicapyridines, pyridine containing polyketides alkaloids which represent additional products of the formicamycin biosynthetic gene cluster, by the aid of targeted metabolomics. The amount of these compounds is at least 100-fold lower than the major compounds, so it is challenging for isolation and

structural identification. Subsequent mutation experiments indicated that the productivity of formicapyridines were increased in the antibiotic biosynthetic monooxygenase decode forS-deletion mutant, while the major products titer was decreased. According to these results, they speculate that ForS had the non-catalytic function but maintaining biosynthetic pathway fidelity in the biosynthetic pathway. While, more experiments need to be done before publication, including some suggestions/questions as following:

We thank the reviewer for their valuable and helpful comments and have done our best to address them below.

1. Title is too vague and broad, which likes a review. Importantly, there are no many works on evolution and engineering, both of which are not sufficiently expressed in the text.

The title has been changed in response to this comment.

2. Cyclases were well discussed, but there is no supportive data and meaningful results in discussion. Although it is interesting that the deletion of forS promotes the production of formicapyridines, there is no further research to be carried out to illuminate its function. The possible biosynthesis of formicapyridine backbone was only proposed, but the transamination was not systematically analyzed.

It is unclear what the reviewer's anticipated response to this comment is. Based on our observations in the WT strain and mutants, and review of the published literature, we formed a hypothesis regarding the potential role of ABM family enzymes in PKS complex formation/function. While further work will be carried out to understand the basis of this functionality, this could take years to achieve. We believe, as reviewers 1 and 2 support, that the data and hypothesis that we forward in this paper will be of great interest to the biosynthetic community and will act as a stimulus for other groups to address our ideas. Moreover, we address the transaminase proposal below in response to question 7.

We have extended our thoughts on the biosynthesis of formicapyridines, in particular the introduction of the nitrogen atom (see above comment to reviewer 1, and manuscript text). The 13C-acetete feeding data is consistent with our pathway hypothesis.

3. The authors mentioned that the stereochemistry of compounds 1-6 were unassigned because of the small amounts and very weak electronic dichroism. Indeed, the productivity of these formicapyridines is very low in WT strain, but the titer was increased approximate 25-fold in the $\Delta$ forS mutant, which provide a chance to determine the stereochemistry. So, I think enough compounds should be obtained to assign the stereochemistry.

We have scaled up production and isolated further material. However, despite this the compounds exhibit very weak optical activity as described and they do not yield ECD spectra from which we can make an atropoisomeric assessment.

4. There are some impurity peaks and peaks with weak signal in NMR data, such as supplementary Figure 18 and supplementary Figure 30. In supplementary Figure 10, there should be more data points (at least 5) to be collected to draw calibration curves. Also, experiments should be repeated three times to obtain error bar.

The 13C NMR spectra in Figs 18 and 30 are indeed weak with poor signal to noise ratios, we had only small amounts of material. However, they are of sufficient quality that with the

additional data from the 2D spectra (HSQC and HMBC) we were able to assign the structures in which we are confident.

The calibration curves were rerun with appropriate replicates and is included in the various supplementary files. Using these we reanalysed the data, which did not change significantly (the changes were within the decimal point). In accordance with this a new version of Supplementary Dataset 2 has been uploaded.

5. Page 15, line 365, please cite references for PKS cyclases as fusion proteins with an ABM domain; in addition, in regard to the function of ABM, is it possible to distinguish chaperone-like function and enzymatic function in nucleotide sequence or amino sequence?

The requested references have been included (27-31). Please note that they are referred to in the section with original line numbers 256-261.

Regarding the function of ABM domain proteins, we performed alignments and phylogenetic analysis of all sequences for characterised ABM monooxygenases, ABM domain fusions with PKS cyclases or any other PKS associated gene, ABM proteins from all of the PKS clusters discussed in the paper [SET 1], and then approx. 100 additional sequences that were the top Blast hits using the ForS sequence as search. All SET 1 sequences contained the conserved active site W66 residue previously identified in the monooxygenase ActVA-orf6 and one other conserved residue. Given the paucity of characterised ABM domain proteins we could make no inferences from the phylogenetic analysis. On the basis of this analysis we have made no additional comments in the manuscript.

6. In Figure 6a and Supplementary Figure 6f, there is a unlabeled peak at ca. 9.8 min, is it related with formicapyridines or formicamycin?

We presume the reviewer means the peak at 9.8 min in Figs 6b-c rather than 6a? The peaks have been labelled with an asterisk and the legend modified to clarify that these compounds are unrelated to the for pathway based on UV and *m/z* analysis.

The major peaks in Supplementary Fig6f correspond to an unrelated metabolite produced variably by *S. formicae* KY5; again, this has been noted in the appropriate figure legend.

7. Is there any gene encoded potential transaminase in for gene cluster or in the genome? If that, overexpression of it may enhance the productivity of formicapyridines.

We have searched the *S. formicae* KY5 genome for transaminase genes and can confirm that there are none in the formicamycin biosynthetic gene cluster. Similarly, there is no gene that would suggest it might be associated with this transformation specifically, but as one would expect there are numerous putative transaminase genes. In line with this analysis we have added text to the relevant section of the manuscript.

8. Minor mistakes: (a) TEXT, in the legend of Figure 3, change “NOSEY (red double head 166 arrows)” to “NOSEY (red double headed 166 arrows)”; (b) in Electronic SI, P40, change “COMPOUND 15 (fasamycin F)” to “COMPOUND 13 (fasamycin F)”; (c) the labels of each chromatogram in Supplementary Figure 9 is not consistent with their legend, please check it again.

These typos have been corrected.

REVIEWERS' COMMENTS:

Reviewer #1 (Remarks to the Author):

The authors of this interesting manuscript have addressed the referee comments on their previous version. This reviewer did not detect any further problems and recommends publication

Reviewer #2 (Remarks to the Author):

The authors corrected/clarified all I asked for. This should be accepted now.

Reviewer #3 (Remarks to the Author):

Now I agree with its publication in Nature Communications.
